# From Capture–Recapture to No Recapture: Efficient SCAD Even After Software Updates

**DOI:** 10.3390/s26010118

**Published:** 2025-12-24

**Authors:** Kurt A. Vedros, Aleksandar Vakanski, Domenic J. Forte, Constantinos Kolias

**Affiliations:** 1 University of Idaho, Moscow, ID 83844, USA; vakanski@uidaho.edu; 2 University of Florida, Gainesville, FL 32611, USA; dforte@ece.ufl.edu

**Keywords:** side-channel analysis, anomaly detection, generative adversarial networks

## Abstract

Side-Channel-based Anomaly Detection (SCAD) offers a powerful and non-intrusive means of detecting unauthorized behavior in IoT and cyber–physical systems. It leverages signals that emerge from physical activity—such as electromagnetic (EM) emissions or power consumption traces—as passive indicators of software execution integrity. This capability is particularly critical in IoT/IIoT environments, where large fleets of deployed devices are at heightened risk of firmware tampering, malicious code injection, and stealthy post-deployment compromise. However, its deployment remains constrained by the costly and time-consuming need to re-fingerprint whenever a program is updated or modified, as fingerprinting involves a precision-intensive manual capturing process for each execution path. To address this challenge, we propose a generative modeling framework that synthesizes realistic EM signals for newly introduced or updated execution paths. Our approach utilizes a Conditional Wasserstein Generative Adversarial Network with Gradient Penalty (CWGAN-GP) framework trained on real EM traces that are conditioned on Execution State Descriptors (ESDs) that encode instruction sequences, operands, and register values. Comprehensive evaluations at instruction-level granularity demonstrate that our approach generates synthetic signals that faithfully reproduce the distinctive features of real EM emissions—achieving 85–92% similarity to real emanations. The inclusion of ESD conditioning further improves fidelity, reducing the similarity distance by ∼13%. To gauge SCAD utility, we train a basic semi-supervised detector on the synthetic signals and find ROC-AUC results within ±1% of detectors trained on real EM data across varying noise conditions. Furthermore, the proposed 1DCNNGAN model (a CWGAN-GP variant) achieves faster training and reduced memory requirements compared with the previously leading ResGAN.

## 1. Introduction

Side-Channel-based Anomaly Detection (SCAD) has recently emerged as a powerful framework for detecting anomalies, integrity violations, and malware in IoT and cyber–physical systems by leveraging unintentional emission leakage such as power consumption, acoustic signatures, and electromagnetic (EM) radiation [1,2]. Rather than relying on a modified software stack, embedded performance counters, and on-chip test–debug infrastructure, SCAD captures these analog signals passively to allow for the remote monitoring of device behavior in real time. In this emerging paradigm, side-channel emissions act as externally observable fingerprints that can validate software integrity without modifying or instrumenting the target device.

Among the alternative side-channel modalities, EM emissions are increasingly favored for passive monitoring in analogous frameworks. Unlike the prevailing alternative of power consumption, which requires intrusive tap points on the target board, EM signals can be captured contactlessly and at a distance, making them ideal for sealed or high-security environments [3,4]. Additionally, the wide frequency spectrum and fast sampling rate enable high-granularity analysis down to the per-assembly instruction level [5]. Recent comparative studies on malware and hardware Trojan detection further show that EM-based approaches not only distinguish benign and malicious executions but also outperform power-based methods in detection accuracy, sensitivity to microarchitectural changes, and resilience to noise [6].

Despite their promise, EM-based SCAD systems face significant scalability and deployment challenges in practice. A core difficulty lies in collecting reliable “fingerprints” of legitimate behavior, which serve as the baseline for anomaly detection. In practice, this fingerprinting process involves several costly and labor-intensive steps: (a) *Manual calibration and expertise-driven alignment* are often required to obtain high-fidelity EM traces under repeatable conditions. (b) *Software with many conditional execution paths* requires exhaustive profiling to ensure that all legitimate branches are covered. (c) *Rare or exception-triggered logic branches* are inherently difficult to activate deterministically during data collection. (d) *Frequent updates or recompilation* of software invalidate existing fingerprints, necessitating repeated EM acquisition and labeling cycles. These practical burdens also explain why a limited number of comparative EM datasets currently exist for SCAD-specific tasks.

**Figure 1:**
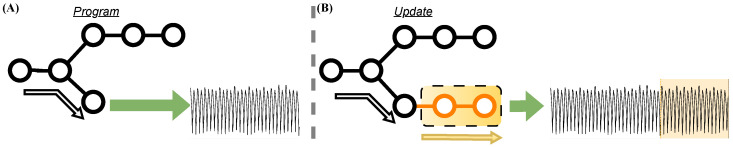
Updated execution path reconstruction. (**A**) presents the preserved original program and EM signal. (**B**) adds the generated EM for the updated code (orange) appended to the original trace.

To address the challenge of maintaining SCAD capabilities following benign software updates, this work focuses on synthesizing realistic EM signals for newly introduced or modified execution paths and enhancing the fidelity of such synthetic emissions through the inclusion of Execution State Descriptor (ESD) information. More specifically, to overcome the bottlenecks associated with manual fingerprinting and the need for repeated EM trace collection due to software updates, we draw upon recent advances in generative modeling—particularly techniques such as voice synthesis using diffusion–GAN hybrids [7] and sketch-to-image translation through adversarial training [8]. *In this context, we propose an approach that employs Generative Adversarial Networks (GANs) to synthesize realistic EM emissions directly from assembly (ASM) code*, thereby eliminating the need for physically recapturing signals for fingerprinting purposes. This concept is illustrated in Figure 1, where prior fingerprinted traces are utilized with synthetic emissions for updated execution paths. Under traditional conditions, any update, no matter how minor, would make manually re-fingerprinting all affected execution branches necessary. However, the proposed hybrid conditioning not only improves signal fidelity but also enables fine-grained adaptation without the need for re-fingerprinting.

This paper makes the following contributions:

- **It proposes a novel generative approach to eliminate the need for re-capturing EM emissions after software updates**, addressing one of the key bottlenecks in SCAD deployment by enabling synthetic signal generation for newly introduced or modified execution paths without requiring physical data re-collection.
- **It introduces Execution State Descriptors (ESDs)** as a novel conditioning mechanism that incorporates prior instructions, operands, and register values, improving the fidelity and contextual accuracy of synthesized EM signals.
- **It presents a 1D-CNN-based CWGAN-GP (1DCNNGAN) architecture** that achieves higher per-instruction fidelity compared with the state-of-the-art ResGAN model while reducing training time by approximately five times and memory usage by an order of magnitude.
- **It validates our approach using per-instruction fidelity evaluation and anomaly detection comparisons**, showing that synthetic EM emissions achieve 82–92% similarity to real signals captured from an Atmel ATmega2560 CPU and yield anomaly detection AUCs nearly equivalent to real-data-trained models.

As this manuscript introduces several technical terms and abbreviations used across the subsequent sections, Table 1 provides a consolidated list of the key acronyms and their corresponding definitions for ease of reference.

The remainder of this paper is organized as follows: Section 2 reviews related work in side-channel-based anomaly detection and generative modeling. Section 3 outlines the system assumptions and threat model of injection attacks. Section 4 introduces the proposed generative framework, detailing the use of CWGAN-GP and Execution State Descriptors. Section 5 outlines the experimental setup. Section 6 presents the evaluation criteria, results, and analysis, and Section 7 contains the overall findings, discusses the broader implications, and outlines future directions.

## 2. Technical Background

SCAD capitalizes on characteristic artifacts within EM emissions that arise from transitions in a processor’s internal register states. Each instruction modifies bits within registers or memory, and these bit flips directly alter current flow through transistors and capacitors—producing unique EM patterns [4,9]. Although many studies examine EM traces at higher-level “block-to-block” transitions (e.g., between loops or function calls) [5,6], such transitions are manifestations of lower-level events such as register updates and bit-state changes occurring beneath the code structure. Anomaly detection using EM signals leverages these distinctive instruction-level emissions as physical fingerprints of computation, enabling deviations in signal patterns to be detected as indicators of anomalous or malicious behavior [6,10,11].

Building a baseline of signals corresponding to the normal states, known as EM fingerprinting, involves collecting large sets of representative traces with precise probe placement and synchronization [6,12]. Even slight changes in instruction order or operand values can affect the EM profile, making it necessary to capture all execution paths [11,13]. Software updates further complicate this, as any modification may require the full re-fingerprinting of affected paths. These challenges render the fingerprinting process labor-intensive and error-prone.

To address these inefficiencies, this work proposes a generative modeling framework capable of synthesizing EM signals for updated or novel execution paths. Inspired by advances in text-to-speech synthesis, we hypothesize that GANs are well-suited for this task due to their proven ability to model complex temporal dependencies and generate high-fidelity, sequence-consistent signals [14,15]. The ResGAN framework [16] represents the state of the art in EM synthesis for SCAD, generating realistic emissions directly from the assembly code and reducing the dependence on physical capture. However, it does not exploit the abundant contextual information already available during program execution—specifically, the state context. Because the state descriptors can be efficiently extracted at runtime, they provide a natural means to preserve signal fidelity and continuity across software versions [17,18]. Yet, existing generative approaches overlook this temporal linkage, treating every update as a wholly new training problem. Consequently, even when large segments of the execution remain unchanged, these models regenerate the entire execution path instead of reusing previously captured traces with identical states up to the update point—ultimately reducing their applicability and robustness.

### 2.1. Generative Adversarial Network (GAN)

A GAN consists of two neural networks—the generator *G* and the discriminator *D*—engaged in a competitive learning process. The generator transforms random noise $z∼p_{z}\left(z\right)$ into synthetic samples G(z) that mimic real data, while the discriminator attempts to distinguish between real data $x∼p_{data}\left(x\right)$ and generated data G(z). Their interaction is modeled as a minimax game:

(1) $$\min_{G}\max_{D}\;E_{x∼p_{\mathrm{data}}\left(x\right)}\left[\log D\left(x\right)\right]+E_{z∼p_{z}\left(z\right)}\left[\log\left(1-D\left(G\left(z\right)\right)\right)\right]$$

where E denotes the expected value. During training, both models iteratively improve—*G* becomes better at fooling *D*, and *D* sharpens its ability to detect fakes. Ideally, this process converges to a Nash equilibrium, where the generator produces realistic, high-fidelity data that the discriminator can no longer reliably distinguish from real samples [14].

### 2.2. Conditional Wasserstein GAN with Gradient Penalty (CWGAN-GP)

The conditional GAN [19] extends the traditional GAN framework by incorporating auxiliary information—such as class labels or feature descriptors *y*—into both the generator and the discriminator. This conditioning guides the generator to produce samples that are not only realistic but also consistent with the specified condition. In a CGAN, the generator receives both random noise *z* and condition *y*, generating samples G(z,y), while the discriminator (or critic) evaluates authenticity based on both the input sample *x* and the condition *y*.

Traditional GANs optimize the Jensen–Shannon (JS) divergence between the real and generated data distributions [20]. However, this objective often provides poor gradient feedback, especially in high-dimensional spaces where the two distributions lie on disjoint manifolds [21]. To overcome these limitations, the Wasserstein GAN (WGAN) reformulates the objective using the Wasserstein (Earth-Mover) distance, replacing the binary discriminator with a continuous-valued critic that estimates this distance. This change improves gradient smoothness, training stability, and convergence [22].

The WGAN framework requires the critic to satisfy the 1-Lipschitz constraint [23], initially enforced through weight clipping [21], which can restrict model capacity and harm convergence. The improved WGAN-GP addresses this by applying a *gradient penalty* to encourage the gradient norm to remain close to unity, thereby maintaining Lipschitz continuity while avoiding mode collapse [22].

Combining these advancements, the CWGAN-GP integrates conditioning with the stability of the WGAN-GP formulation [19,24]. This design allows the generator to produce condition-specific, high-fidelity outputs with stable convergence even in complex domains such as EM signal synthesis. The CWGAN-GP objective is defined as

(2) $$\underset{\mathrm{Wasserstein}\;\mathrm{Loss}}{\underset{︸}{\frac{1}{N}\sum_{i=1}^{N}\left[D\left(x_{i}|y_{i}\right)-D\left(G\left(z_{i},y_{i}\right)|y_{i}\right)\right]}}+\underset{\mathrm{Gradient}\;\mathrm{Penalty}}{\underset{︸}{\lambda \frac{1}{N}\sum_{i=1}^{N}{\left(∥\nabla_{\hat{x}}D\left({\hat{x}}_{i}|{\hat{y}}_{i}\right){∥}_{2}-1\right)}^{2}}}$$

where $x_{i}$ are real samples, $z_{i}$ are latent noise vectors, $y_{i}$ are conditioning variables, ${\hat{x}}_{i}$ are interpolated samples between real and generated data, and λ controls the strength of the gradient penalty. Together, these components ensure that the generator learns condition-dependent mappings with improved training robustness and realistic data synthesis fidelity.

### 2.3. Tokenizer

In the proposed framework, tokenization [25] is applied only to the textual component of the Execution State Descriptor (ESD)—specifically, the ASM instruction mnemonics—because the generative model cannot directly process raw words or strings. Each mnemonic is mapped to a unique integer index, producing a compact and machine-readable representation that preserves instruction identity, as shown in Equation (3). By contrast, the other elements of the ESD—namely, operands, register values, and cycle indices (see Section 4)—are inherently numerical and thus can be provided to a generative model (GM) unchanged.

(3) $$\mathrm{Tokenize}:\;\left\{m_{i},m_{i+1},\ldots ,m_{i+n}\right\}\;⟷\;\left\{t_{i},t_{i+1},\ldots ,t_{i+n}\right\}$$

Unlike modern natural language processing pipelines that increasingly rely on sophisticated word-embedding techniques [26,27], the proposed framework intentionally adopts a minimal tokenization strategy. Although embeddings can encode richer relationships such as instruction co-occurrence patterns or likely successor relationships, we found no empirical need for such complexity; the GM operates on deterministic execution traces where instruction ordering and operand patterns are already explicitly represented within the ESD, as discussed in Section 4.1.1. In practice, this base tokenization approach provides sufficient representational capacity for accurate EM synthesis while maintaining transparency and reproducibility.

## 3. Assumptions and Threat Model

To ensure realistic evaluation of the proposed framework, this section outlines the underlying system assumptions and threat model considered throughout this work. We begin by describing the operational context in which EM-based monitoring is performed and the architectural characteristics that make the target platform suitable for instruction-level modeling. We then explain the rationale behind partial trace synthesis and the choice of assembly as the input representation, followed by the assumptions governing the attacker’s capabilities and operating environment.

### 3.1. Operational Context

In this work, the device was originally placed under EM-based monitoring and manually fingerprinted: representative EM traces were collected, aligned, and execution paths identified to form the baseline used for anomaly detection. Since that initial fingerprinting, software updates (benign patches or feature changes) have been applied to the program, producing new or modified execution paths that are not covered by the original baseline. Consequently, the system would normally require the re-fingerprinting of affected paths to restore detection coverage, a process that is costly and time-consuming and is the principal operational challenge that this paper seeks to mitigate.

### 3.2. IIoT Microcontroller/Embedded CPU Assumptions

The proposed generative modeling approach is particularly advantageous for the IIoT due to its simple, deterministic software compared with multi-threaded CPUs. Microcontrollers such as the Atmel AVR used in this study lack complex features like caches, speculative execution, or interrupt-driven scheduling, minimizing confounding factors and improving trace consistency. Their single-task, real-time behavior ensures clean EM captures, which are critical in domains such as automotive systems, medical devices, and industrial controllers, where predictable performance and low latency are essential [28,29]. These characteristics enable the precise instruction-level modeling of side-channel emissions using GANs trained on compact, context-rich descriptors.

### 3.3. Practicality of Synthesizing Only the Updated/Modified Segments

Rather than synthesizing an entire program trace, the framework limits synthesis to newly introduced or modified segments for both fidelity and practicality. ESDs for instructions preceding an update are readily available from the pre-update baseline (obtained during initial fingerprinting or by running/emulating the prior code [17,18]), and ESDs for the new segment can be derived deterministically via static inspection, emulation, or the instruction-level tracing of the assembly. These ESDs (instruction sequence, operands, and register values) provide the contextual state that directly shapes EM morphology and—when included—substantially improve generated-signal fidelity. In contrast, full-path generation lacks an explicit representation of prior execution states, forcing the model to depend solely on the instruction sequence. Moreover, when the system has already been fingerprinted, the pre-update emissions are genuine physical captures and thus have perfect fidelity; reusing those real segments and generating only the changed portion avoids propagating minor generative errors across unchanged regions and is therefore both more accurate and more efficient.

### 3.4. Assembly (ASM) as the Chosen Input Representation

ASM code serves as an ideal input representation for this process for several reasons. (1) It provides direct visibility into the actual machine-level operations that drive EM leakage. (2) Nearly all high-level languages, including C and Arduino sketches, are compiled down to assembly, making it broadly applicable across platforms. (3) Assembly code is easily obtainable from compiled binaries through standard disassembly tools. (4) Finally, its one-to-one mapping between instructions and processor activity allows for the fine-grained, instruction-level modeling of EM emissions.

### 3.5. Attacker Capabilities and the Experimental Threat Model

We make the following assumptions about the attacker’s capabilities and the experimental threat model. An informed adversary may analyze the target binary or exploit a vulnerability (for example, via buffer overflow or code injection) to insert, delete, or modify code; in general, such an attacker could effect any number of changes. However, to evaluate stealthy, worst-case evasion scenarios, our experiments consider an adversary who elects to make the smallest plausible modification that still achieves a malicious objective (e.g., inserting, removing, or altering a single instruction). This choice reflects a realistic tradeoff: smaller changes are harder to detect via side-channel fingerprints, whereas more extensive modifications tend to produce more conspicuous deviations in execution behavior and thus are easier to detect by SCAD approaches. Finally, we assume that the attack occurs in an environment representative of normal deployment (i.e., not a noise-free lab). Ambient and operational noise may degrade signal clarity and must be accounted for in both capture and synthesis; therefore, our detection evaluations do not rely on lab pristine traces.

## 4. Proposed Framework

**Figure 2:**
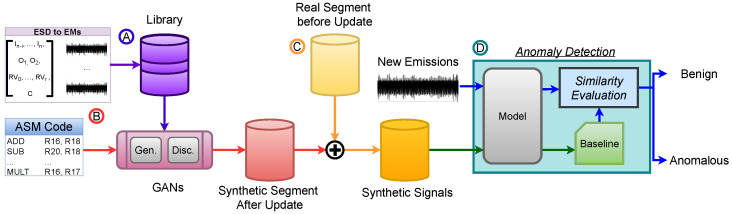
Overview of the proposed framework: Ⓐ ESD to EM signal library, Ⓑ CWGAN-GP–based EM synthesis, Ⓒ reconstruction of the updated EM trace, and Ⓓ anomaly detection.

The proposed framework integrates code analysis, generative modeling, and anomaly detection into a unified pipeline for reconstructing and evaluating EM emissions after software updates. As shown in Figure 2, the process begins with a library of Execution State Descriptors (ESDs) and their corresponding EM signatures Ⓐ, which serve as the foundation for conditional signal synthesis. The CWGAN-GP model then trains on this library and generates EM emissions for new or modified assembly instructions according to the new update program after the ESD representations are extracted Ⓑ. These generated segments for each ESD are assembled in the updated program order and combined with the preserved portions of the previously fingerprinted trace to produce full synthetic EM signals that reflect the updated code execution Ⓒ. Finally, the resulting synthesized traces populate the updated baseline used by the anomaly detection module, which compares runtime emissions against the new reference behavior Ⓓ. A detailed explanation of each stage (A–D) is provided in the following subsections.

### 4.1. Library

The framework assumes that a *library of basic building blocks* that can be used to train the generative models (GMs) exists. In this work, the goal is to detect malicious modifications with “per instruction” accuracy. Therefore, in this context, “building blocks” correspond to ASM instructions (of a given CPU) mapped to corresponding EM signals, as shown in Figure 2Ⓐ. Notably, this library only needs to be generated once per CPU architecture, as future GMs can be trained using the same dataset. However, constructing a library that faithfully captures the realistic EM morphology across device states and operating conditions remains challenging.

#### 4.1.1. The Impact of Preceding Instructions

A naïve approach [30] would be to collect EM signals from individual instructions executed in dummy programs, isolating each with surrounding nop operations. However, this method overlooks critical factors such as microarchitectural features of CPUs. For example, CPUs like the Atmel AVR employ a two-stage pipeline (fetch and execute), causing instructions to overlap in execution. As a result, an instruction’s EM signature is shaped not only by itself but also by neighboring instructions. This necessitates modeling instruction patterns in at least pairs, rendering the simplistic fingerprinting of isolated instructions inadequate for capturing realistic execution behavior.

**Figure 3:**
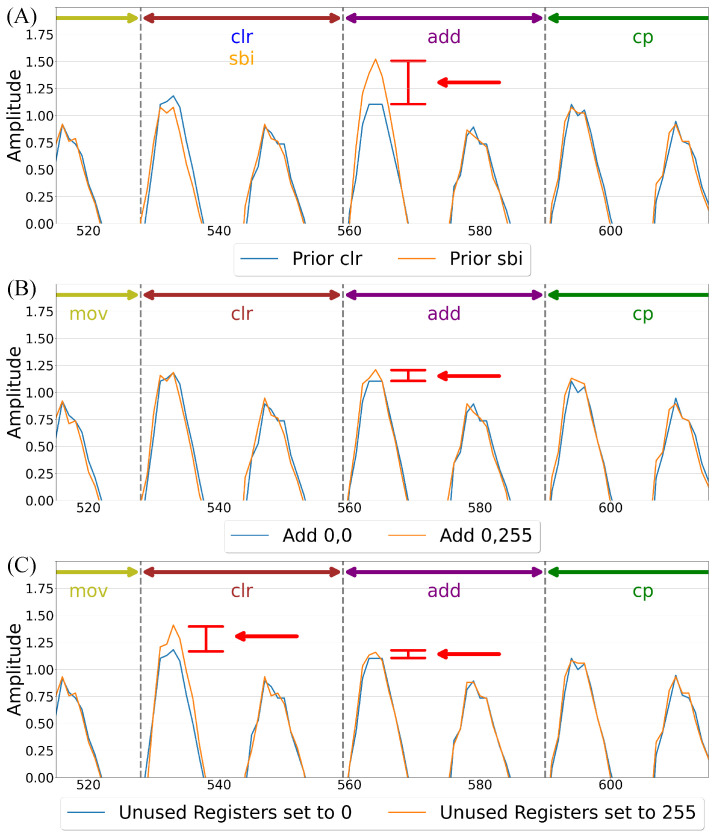
Influence of state on EM morphology: (**A**) depicts prior instruction influence; (**B**) showcases operand effects; and (**C**) shows the impact of unused register values, highlighted by red arrows.

Furthermore, prior instructions significantly influence a device’s EM emissions during the execution of a given ASM instruction, as illustrated in Figure 3A, where the add instruction’s first peak differs notably depending on whether it is preceded by a clr or sbi instruction. This variation stems from residual electrical charges and transient states left by preceding operations, which influence the device’s internal conditions [16,31]. When an instruction executes, it alters the distribution of electrical charges across components like capacitors and transistors. These charges persist briefly and can affect the EM signature of the instructions that follow. Thus, sequences of instructions $I_{n-i},\ldots I_{n}$ must be considered to accurately capture inter-instruction dependencies. Moreover, because each instruction may span multiple execution cycles, it is necessary to determine which specific cycle *C* is portrayed in the corresponding emissions.

#### 4.1.2. Accounting for State Transitions

In this context, the term *device state* refers to the internal conditions present during execution, including register values, flag statuses, and electrical characteristics like capacitor charge levels. These factors significantly impact the morphology of EM signals generated by instructions. For example, instructions involving bit flips—changes in registers, flags, or memory—produce higher EM amplitude due to the energy required to switch bit states, even if the instruction itself remains unchanged.

While accounting for all possible device states would be ideal, it is practically infeasible due to the vast number of combinations. However, a practical approximation is possible. We hypothesize that most state-dependent EM variation can be captured by focusing on two main factors: (1) *the operands being used* and (2) *the current register values before execution* (i.e., current state).

**Operands:** Operands influence the EM signal of an ASM instruction by altering the device’s internal state, as the operation requires electrical energy to update memory and registers. This is evident in Figure 3B, where the add instruction’s first peak shifts slightly depending on whether it adds 0 to 0 or 0 to 255. During execution, bit transitions cause charge redistribution across transistors and capacitors, and the number and pattern of these flips directly impact EM emissions. This effect is particularly noticeable in arithmetic and logical operations, where varying operand values lead to different transition patterns, resulting in fluctuations in EM amplitude and frequency spectra [32,33]. The operands are given as $O_{1}$ and $O_{2}$ in Figure 2Ⓐ.

**Register Values:** Current register values during execution influence a device’s EM emissions, as shown in Figure 3C, where changing register values not used in the instruction from 0 to 255 heavily impacts the clr instruction and even slightly alters the morphology of the subsequent instruction in this case, add. This effect arises from both the dynamic activity of bit transitions during execution (see Section 4.1.2) and the static power required to maintain register states. Even when values remain unchanged, static power is consumed due to leakage currents inherent in semiconductor devices [34]. This continuous power draw contributes to the EM signature, with each maintained bit requiring a small but steady current. Studies indicate that static power dissipation accounts for an increasing share of total power consumption in modern semiconductor technologies [34,35]. The number of registers *r* is determined by the device and is represented as $RV_{0},\ldots ,RV_{r}$ in Figure 2Ⓐ.

**ESD–EM Library Construction:** To capture instruction-level execution behavior across relevant state transitions, the framework maintains a library that maps ESDs to collections of corresponding EM emission signals. Each entry in the library consists of an ESD–EMs set pair $\left({\mathrm{ESD}}_{k},S_{k}\right)$, where *k* indexes are the amount of distinct execution states observed during training.

To capture instruction-level execution behavior across all relevant state transitions, the framework maintains a library that maps ESDs to their corresponding EM emission segments, as summarized in Equation (6). Each entry in the library consists of an ESD–signals pair (${\mathrm{ESD}}_{k}$,$S_{k}$), where *k* indexes distinct execution states observed during training.

An ESD encodes the local execution context of a target instruction and is defined as

(4) $${\mathrm{ESD}}_{k}=\left[I_{n-i},\ldots ,I_{n},O_{1},O_{2},RV_{o},\ldots ,RV_{r}\right]$$

where $I_{n-i},\ldots ,I_{n}$ denote the sequence of preceding and matching assembly instructions required to capture pipeline overlap and residual-state effects. The operands $O_{1},O_{2}$ correspond to the two explicit operands supported by the AVR instruction set; note that although we indicate this by showing two values $O_{1},O_{2}$, the operand values associated with each of the preceding and matching instructions are taken into account in the same order as the instructions obtained prior. Finally, $RV_{o},\ldots ,RV_{r}$ represent the values contained inside the registers immediately prior to execution, capturing the device’s internal state that influences EM morphology.

Each ESD is associated with a *set* of EM emission signals defined as

(5) $$S_{k}=\left\{\;s_{1},s_{2},\ldots ,s_{j}\;\right\},$$

where each $s_{j}$ is a complete EM signal corresponding to a single execution cycle of the instruction described by ${\mathrm{ESD}}_{k}$. Multiple signal realizations are included to capture intra-state variability arising from measurement noise, minor timing variations, and environmental effects.

Collectively, the library forms a structured mapping from execution states to physical emissions that serves as the conditioning dataset for the CWGAN-GP model, and can be expressed compactly as

(6) $$\left[\begin{array}{c} {\mathrm{ESD}}_{1}\\ {\mathrm{ESD}}_{2}\\ \vdots \\ {\mathrm{ESD}}_{k} \end{array}\right]\;\to \left[\begin{array}{c} S_{1}\\ S_{2}\\ \vdots \\ S_{k} \end{array}\right]$$

### 4.2. CWGAN-GP Architecture

The proposed framework leverages GANs to synthesize high-fidelity EM signals from structured Execution State Descriptors (ESDs) that include instruction sequences, operands, and register value information, as shown in Figure 2Ⓑ. By operating at the ASM level, the framework also achieves scalability across diverse programming paradigms, as all high-level languages ultimately compile to ASM.

**Figure 4:**
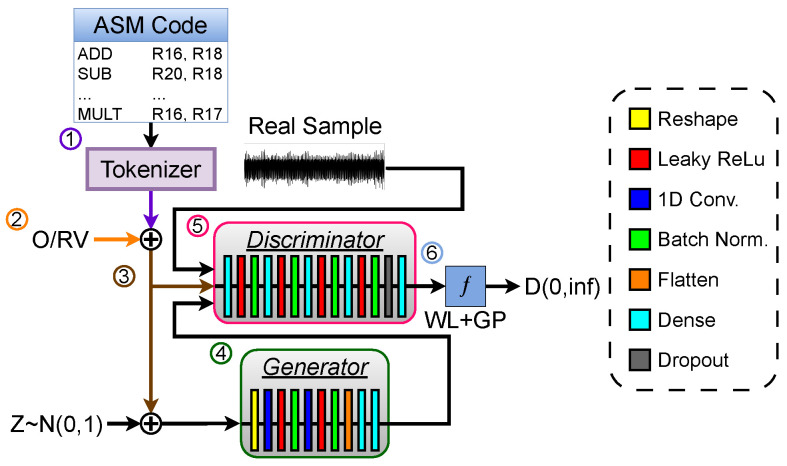
Proposed CWGAN-GP workflow: ① tokenized ASM code, ② Operands and Register Values added to create the ESDs, ③ ESDs sent to the Discriminator and Generator with latent noise, ④ Generator, ⑤ Discriminator, and ⑥ Wasserstein loss and gradient penalty.

In further detail, as illustrated in Figure 4, the proposed CWGAN-GP workflow uses a tokenization strategy that encodes the device’s ESDs at the assembly level, allowing for learning from programs of varying lengths ①. Each ASM instruction is mapped to a token, with operands, register values at execution, and the target cycle appended ②. This structured input captures both instruction sequence and system state, improving the model’s generalization to diverse instruction patterns. A CGAN framework is employed to synthesize EM emissions across multiple instruction types and states within a single model, avoiding the need for separate GANs per program ③. By feeding sequences of ASM instructions in their original binary order, the model accounts for residual charge effects and preserves instruction-level dependencies, enabling realistic EM signal generation. The generator ④ and discriminator ⑤ in the framework operate together to synthesize realistic EM emissions from instruction sequences while distinguishing real from generated signals.

To improve training stability and the fidelity of generated EM signals, the framework integrates WGAN-GP ⑥, a key enhancement over traditional GANs. Standard GAN loss functions often suffer from mode collapse and vanishing gradients, especially when generating high-dimensional signals like EM waveforms. Since our model must produce realistic emissions across diverse instruction sequences, stable training and accurate gradients are essential. WGAN-GP enhances stability by replacing the standard adversarial objective with the Wasserstein distance and by introducing a gradient penalty term that enforces the 1-Lipschitz constraint required for the critic. The WGAN-GP loss function is provided in Equation (7) below with each component annotated:

(7) $$\begin{array}{cc} L_{\mathrm{WGAN-GP}}= & \underset{\mathrm{Wasserstein}\;\mathrm{loss}}{\underset{︸}{E_{x∼P_{r}}\left[D\left(x\right)\right]-E_{z∼P_{z}}\left[D\left(G\left(z\right)\right)\right]}}\\ \; & +\underset{\mathrm{Gradient}\;\mathrm{penalty}}{\underset{︸}{\lambda \;E_{\hat{x}∼P_{\hat{x}}}\left[{\left({∥\nabla_{\hat{x}}D\left(\hat{x}\right)∥}_{2}-1\right)}^{2}\right]}} \end{array}$$

While generative modeling in domains such as text-to-speech synthesis increasingly favors autoregressive, diffusion, or transformer-based architectures, CWGAN-GP remains the most suitable choice for the EM signal synthesis task explored in this work. Variational autoencoders (VAE), while computationally efficient and stable to train, typically struggle to reproduce the fine-grained temporal morphology required for high-fidelity instruction-level EM synthesis. Unlike autoregressive models, which require sequential sample-by-sample generation and incur significant latency, CWGAN-GP generates entire EM segments in parallel—an important property given the thousands of traces required to build synthetic cycle libraries. Diffusion and transformer models, although capable of exceptional fidelity, introduce substantial computational overhead, require large training datasets, and rely on long-range attention or iterative denoising processes. In contrast, CWGAN-GP provides stable gradient behavior, strong mode coverage, and efficient conditional generation, making it well-suited for capturing the fine-grained morphological variations driven by instruction context and device state. Furthermore, the Wasserstein objective with gradient penalty offers robustness when learning from limited and noisy EM datasets [22,24]. For these reasons, CWGAN-GP offers the best balance of stability, efficiency, and representational fidelity for the targeted application of instruction-conditioned EM synthesis. Table 2 further summarizes the reasoning behind the choice of CWGAN-GP.

Beyond the training formulation itself, the CWGAN-GP framework remains flexible with respect to the internal architectures of its generator and discriminator. In this work, two implementations are explored: **ResGAN**, representing the prior state-of-the-art design that leverages residual connections for gradient preservation and feature reuse, and the proposed **1DCNNGAN**, a lightweight convolutional variant optimized for time-series EM synthesis. The following subsections describe how each model operationalizes this framework—detailing how temporal features are extracted, how conditioning information is fused into the generative pathway, and how architectural choices influence fidelity and stability. For completeness and reproducibility, a full layer-by-layer specification of both models—including token embedding format, layer sizes, convolutional kernel dimensions, activation functions, and training hyperparameters—is provided in Appendix B.

#### 4.2.1. 1DCNNGAN

The 1DCNNGAN architecture employs one-dimensional convolutional (1D CNN) layers coupled with Leaky ReLU activations to efficiently capture the temporal dependencies and localized waveform features characteristic of EM signals. The generator uses a 1D CNN backbone to extract hierarchical temporal features from tokenized input sequences, progressively transforming latent noise and conditioning vectors into fine-grained EM waveforms. Convolutional and transposed-convolutional layers capture both local oscillation patterns and long-range temporal dependencies, while Leaky ReLU activations mitigate vanishing gradients and preserve subtle amplitude variations. Batch normalization is applied after each convolutional block to stabilize gradient flow and improve generalization across diverse instruction states. The final dense layers reshape the output to match the expected EM signal length, yielding continuous, instruction-level emission patterns.

To formally express the synthesis process, the generator $G_{\theta }$ in the proposed 1DCNNGAN maps a latent vector *z* and an Execution State Descriptor (ESD) *y* into a continuous one-dimensional EM waveform $\tilde{x}$. This generative mapping can be expressed as

(8) $$\tilde{x}=G_{\theta }\left(z,y\right)=\mathrm{Reshape}\;(W_{2}\;\phi_{\mathrm{ReLU}}\;\left(W_{1}\;\mathrm{Flatten}\;\left({\mathrm{BN}}_{2}\;\left(\phi_{\mathrm{LReLU}}\;\left(\mathrm{Conv}1D_{2}\;\left({\mathrm{BN}}_{1}\;\left(\phi_{\mathrm{LReLU}}\;\left(\mathrm{Conv}1D_{1}\left(\left[z,y\right]\right)\right)\right)\right)\right)\right)\right)\right))$$

where z∼N(0,I) represents the latent noise, *y* encodes the instruction, operand, and register-state information; $\mathrm{Conv}1D_{i}$ and ${\mathrm{BN}}_{i}$ denote the $i^{\mathrm{th}}$ convolutional and batch-normalization layers; $\phi_{\mathrm{LReLU}}\left(\cdot \right)$ and $\phi_{\mathrm{ReLU}}\left(\cdot \right)$ represent Leaky ReLU and ReLU activations, respectively; $W_{1}$ and $W_{2}$ are dense projection matrices; and the final reshape operation produces the one-dimensional waveform output.

The discriminator follows a fully connected architecture that learns to distinguish real and synthetic EM traces through a sequence of dense layers employing Leaky ReLU activations, batch normalization, and dropout. This configuration enables the discriminator to model complex nonlinear relationships while preventing overfitting and maintaining adversarial stability. A final sigmoid activation outputs a probability score representing the authenticity of each signal, providing feedback that guides the generator toward increasingly realistic EM synthesis.

#### 4.2.2. ResGAN

The ResGAN [16] architecture serves as the baseline generative model within the proposed framework, combining residual learning with convolutional and fully connected layers to generate high-fidelity EM signals from tokenized assembly instructions. The generator begins by reshaping its input into a three-dimensional tensor to enable convolutional processing. A single one-dimensional convolutional layer follows, replicating the input features across the new dimension and enabling the extraction of localized temporal dependencies within instruction sequences. The resulting three-dimensional feature map is passed into a ResNet-50 backbone that applies stacked residual blocks, where each block computes F(x)+x to preserve gradient flow and reinforce information learned from prior layers. This residual learning strategy allows the network to model both fine-grained oscillation patterns and long-range amplitude dependencies in EM waveforms. The ResNet output is reduced using global average pooling to form a compact feature representation, followed by a dropout layer to minimize overfitting and encourage generalization. Finally, a series of dense layers projects the learned representation into a one-dimensional output of fixed length corresponding to the EM waveform.

Formally, the generator $G_{\phi }$ in the ResGAN model transforms a latent vector *z* and conditioning token sequence *y* into a synthetic EM waveform $\tilde{x}$. The overall mapping can be expressed as

(9) $$\tilde{x}=G_{\phi }\left(z,y\right)=W_{4}\;\phi_{3}\;\left(W_{3}\;\phi_{2}\;\left(W_{2}\;\phi_{1}\;\left(\mathrm{GAP}\;(\sum_{k=1}^{K}\left[F_{k}\left(h_{k-1}\right)+h_{k-1}\right])\right)\right)\right)$$

where z∼N(0,I) is the latent input and *y* encodes the tokenized instruction sequence. Each $F_{k}\left(\cdot \right)$ denotes the $k^{\mathrm{th}}$ residual transformation in the ResNet backbone (comprising convolutional, batch-normalization, and ReLU layers), and $h_{k-1}$ is its input feature map. The summation captures residual accumulation across all *K* blocks. GAP(·) is global average pooling, and $\phi_{i}\left(\cdot \right)$ denotes ReLU activations applied after successive dense transformations $W_{i}$ (i=1, 2, 3, 4), with $W_{4}$ producing the final one-dimensional waveform output $\tilde{x}$.

The discriminator in the ResGAN design follows a simpler configuration composed exclusively of fully connected layers. Each dense layer applies a standard ReLU activation to model nonlinear relationships within the input signal space, culminating in a final sigmoid activation that outputs a scalar authenticity probability.

### 4.3. GAN Training

The training process follows the CWGAN-GP framework. It begins by extracting an Execution State Descriptor (ESD) for each instance, which encapsulates the matching and preceding instructions, their operands, the register values prior to execution, and the clock cycle associated with the target instruction. The instruction pair is tokenized as $t=\phi (\left[i_{n-1},i_{n}\right])$ and combined with the remaining ESD components. A latent noise vector z∼N(0,I) is then sampled from the Gaussian distribution and concatenated with the full ESD representation. This composite vector is passed to the generator *G*, which synthesizes an EM waveform conditioned on the encoded execution state.

The generated signal $\tilde{x}=G\left(z,esd\right)$ and a real signal *x* with the same ESD are then passed to the discriminator *D*. The discriminator evaluates the consistency of each signal–ESD pair, distinguishing authentic EM emissions from synthetic ones. Following the CWGAN-GP formulation, the adversarial objective with gradient penalty is defined as

(10) $$\min_{G}\max_{D}\;E_{x∼P_{r}}\;\left[D(x,esd)\right]-E_{z∼N(0,I)}\;\left[D(G(z,esd),esd)\right]+\lambda \;E_{\hat{x}∼P_{\hat{x}}}\;\left[{\left(∥\nabla_{\hat{x}}D\left(\hat{x},esd\right){∥}_{2}-1\right)}^{2}\right]$$

where $P_{r}$ and $P_{\hat{x}}$ represent the real and interpolated signal distributions, respectively, and λ controls the strength of the gradient penalty enforcing the 1-Lipschitz constraint on *D*.

During training, the discriminator and generator are updated iteratively according to their respective loss gradients. The discriminator is optimized to maximize the Wasserstein distance while maintaining smooth gradients, whereas the generator minimizes the discriminator’s ability to distinguish synthetic signals. These update steps are expressed as

(11) $$\begin{array}{cc} Loss_{D} & =-E_{x∼P_{r}}\left[D\left(x,esd\right)\right]+E_{z∼N(0,I)}\left[D\left(G\left(z,esd\right),esd\right)\right]+\lambda \;E_{\hat{x}}\;\left[{\left(∥\nabla_{\hat{x}}D\left(\hat{x},esd\right){∥}_{2}-1\right)}^{2}\right],\\ D & \leftarrow D-\alpha \nabla_{D}Loss_{D},\\ Loss_{G} & =-E_{z∼N(0,I)}\left[D\left(G\left(z,esd\right),esd\right)\right],\\ G & \leftarrow G-\alpha \nabla_{G}Loss_{G}, \end{array}$$

where α denotes the learning rate. The process repeats across multiple mini-batches, with the discriminator being typically updated several times per generator step to stabilize convergence. Through this iterative adversarial optimization, the generator progressively learns to synthesize EM signals that exhibit high fidelity and statistical consistency with real emissions conditioned on the underlying instruction states.

### 4.4. Synthesizing EM Emissions

As shown in Figure 2Ⓒ, the proposed process for reconstructing a full EM trace for the updated execution path reuses the already-fingerprinted portion of the base trace and synthesizes only the EM emissions corresponding to the newly introduced or modified instructions. The updated program is first analyzed at the ASM level to identify the update segment(s). For each instruction in the update segment, the ESDs, which include the instruction token(s), operands, register values prior to execution, and the target cycle index, are derived. Importantly, the ESDs explicitly incorporate a short window of preceding instructions to capture pipeline overlap and residual-state effects; for boundary instructions at the beginning of the update, these preceding instructions include those in the base program immediately before the insertion point, ensuring continuity and avoiding a context mismatch.

**Figure 5:**
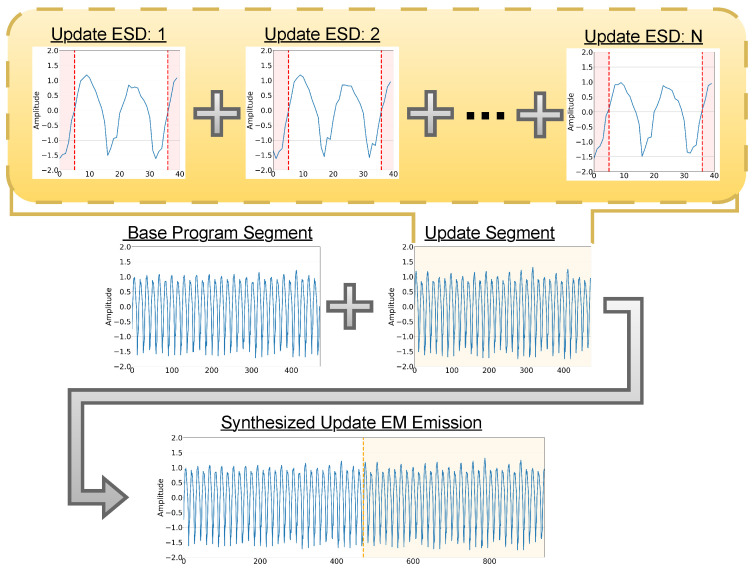
Synthesis of the updated EM trace, where red regions denote removed portions and orange indicates the update segment.

The trained conditional generator then synthesizes an EM waveform snippet for each ESD in the update segment. Note that rather than generating entire long traces directly, the proposed method generates instruction-cycle-level waveforms with a small margin of time-indexed samples before and after the cycle to support robust concatenation. As illustrated in Figure 5, the update segment is then assembled in program order by stitching successive generated cycle waveforms at a low-information boundary: specifically, each cycle is aligned to a near-zero-amplitude crossing and trimmed of the excess samples so that each appended unit begins slightly above 0 and ends close to or at 0. Because the near-zero crossing contains minimal instruction-discriminative information compared with the peak morphology, stitching at this point reduces discontinuities while preserving the informative portions of each cycle. Finally, we take the real base-program EM trace up to the update point and stitch it to the fully assembled synthetic update-segment trace using the same near-zero-crossing rule, producing a complete reconstructed trace for downstream baseline construction and anomaly detection.

### 4.5. Detection Model

Finally, an anomaly detection model is trained on the synthetic samples, and new emissions are evaluated during runtime, as shown in Figure 2Ⓓ. The proposed framework is designed to accommodate any anomaly detection model for EM-based detection, with the key requirement that it follows a semi-supervised approach rather than a fully supervised one. A semi-supervised method is necessary because the base code can undergo near-infinite modifications, making it impractical to rely on traditional supervised learning, which requires labeled examples for every possible variation. However, during normal operation, the number of possible variations remains finite, allowing for the establishment of a baseline of normalcy against which anomalies can be detected.

EM-based anomaly detection can be implemented using either shallow machine learning techniques or deep learning models. While more sophisticated anomaly detection models could yield superior performance, our focus is on generating high-fidelity synthetic signals and evaluating the performance of using such a dataset rather than optimizing anomaly detection accuracy. As such, we opt for a simple Euclidean distance-based semi-supervised kNN approach for our experimentation.

## 5. Experimental Setup

The following section details the test environment and datasets employed during experimentation.

### 5.1. Testbed

**Figure 6:**
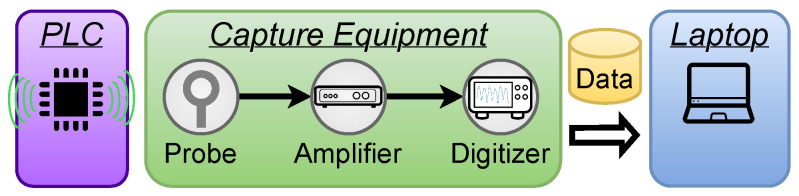
EM acquisition setup used for data collection.

Figure 6 illustrates the testbed for our experiments. The target device was an Arduino Mega, which features an 8-bit ATmega2560 AVR microcontroller unit (MCU) (Arduino S.r.l., Monza, Italy). During program execution, the microcontroller naturally emits EM signals, which were captured using an EMRSS RF Explorer H-Loop EM probe (EMR Shielding Solutions, Austin, TX, USA) positioned directly above the CPU. Since these emissions have very low amplitude, a Behive 150A EMC probe amplifier (Behive Electronics, Sebastopol, CA, USA) was employed to amplify the signal for better analysis. Finally, the acquisition and digitization of the EM signals was performed using a PicoScope 3203D oscilloscope (Pico Technology, Tyler, TX, USA), and the data were sent to and processed on a laptop.

### 5.2. Base Program

For our experiments, we developed a custom program, referred to as Base for simplicity, which serves as the reference program before any modifications are applied. It is assumed that the EM signals corresponding to the execution of the Base program have already been captured and stored.

The Base program consists of 28 assembly instructions and performs a straightforward arithmetic computation:

(12) $$Z=\frac{D}{(A\times B)+C}$$

where the variables *A*, *B*, *C*, and *D* are stored in registers r16, r17, r18, and r19, respectively, while the computed result *Z* is stored in register r20 (Listing 1).

**Listing 1:** Base program’s assembly code.

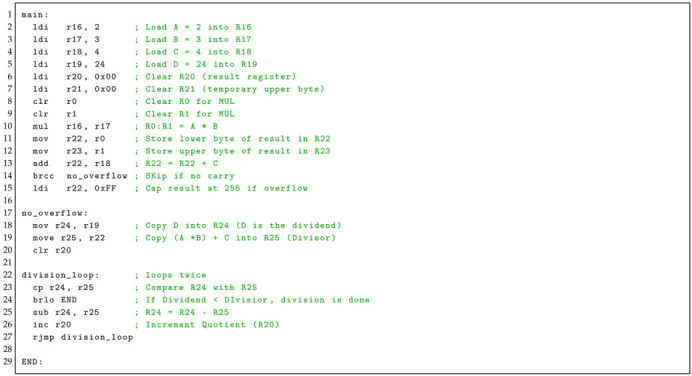



An arithmetic computation was specifically chosen due to its simplicity and the feasibility of executing realistic code without introducing unnecessary complexity such as loop calls.

### 5.3. Update Programs

To evaluate our approach to generate highly accurate synthetic samples, we applied three distinct updates to the *Base* program. These updates, referred to simply as *A*, *B*, and *C*, introduce additional instructions that modify the program’s functionality while preserving its original execution flow. Each update consists of a different number of instructions and performs a unique arithmetic computation.

**Update A** contains six ASM instructions that compute a basic multiplication operation:

(13) Z=A×B

Update A introduces a minimal arithmetic extension that computes the multiplication Z=AB, as shown in Listing 2. The routine begins by clearing registers R0 and R1 to ensure a deterministic execution state, as these registers are implicitly used by the AVR multiply (mul) instruction (lines 2 and 3). Register R22 is also cleared to prepare for storing the final result (line 4). The multiplication of operands *A* and *B* is performed using mulr16, r17, which produces a 16-bit result across registers R0:R1 (line 5). The lower byte of the product is then moved into R22 using movr22, r0, as only the least significant byte is required for subsequent processing (line 6). Execution then jumps directly to the end of the program, bypassing the remaining base code (line 7).

**Listing 2:** Update A’s assembly code. Calculates Z = A× B after a point in the base program’s code.

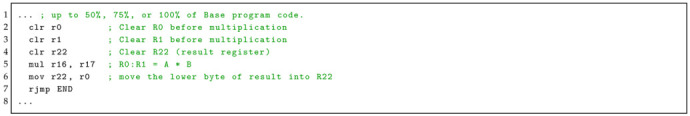



**Update B** expands the complexity with eleven ASM instructions to calculate:

(14) Z=(C×D)−(A×B)

In specifics, update B introduces an eleven-instruction arithmetic expansion that computes the expression Z=(C×D)−(A×B) using AVR assembly operations, as shown in Listing 3. The routine begins by clearing registers R22, R0, and R1 to establish a known execution state, where R22 is reserved for the final result and R0–R1 are implicitly used by the mul instruction (lines 2–4). The product AB is first computed using mulr16, r17, with the lower byte of the result stored in R23 (lines 7 and 8). The routine then computes CD via mulr18, r19, storing the lower byte of this product in R24 (line 11 and 12). The subtraction step is performed using subr24, r23, yielding (C×D)−(A×B) (line 15). A conditional branch (brcc) checks for borrow; if a borrow occurs, the result is saturated to zero using ldir24, 0, ensuring non-negative output (lines 16, 20 and 21). Finally, the computed result is moved into R22 and execution resumes at the end of the update block (line 17).

**Listing 3:** Update B’s assembly code. Calculates *Z* = (*C* × *D*) − (*A* × *B*) after a point in the base program’s code.

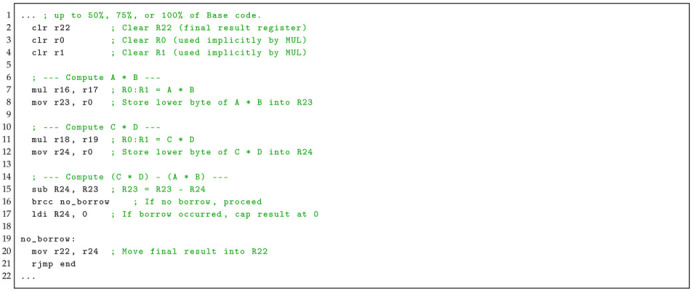



**Update C** is the most complex modification, consisting of fifteen ASM instructions. Before performing its computation, the program checks whether the value stored in register 19 (representing variable *D*) is greater than 20. If this condition is satisfied, the update computes the following expression:

(15) $$Z=\left(C\times D\right)-\left(A^{2}\times B^{2}\right)$$

If the condition is not met, the execution skips the update and continues with the original Base program.

Update C introduces a conditional execution path that selectively performs a higher-complexity arithmetic computation depending on the runtime value of operand *D*, as shown in Listing 4. The routine begins with a comparison instruction (cpir19, 20) that evaluates whether *D* exceeds a fixed threshold, followed by a conditional branch (brloskip_C) that bypasses the update when the condition is not met, allowing execution to continue along the original base program path (lines 3 and 4). When the condition holds, the update computes the product C×D using mulr18, r19, storing the low and high bytes of the result in registers R21 and R22, respectively (lines 7–9). The computation then squares operand *A* via mulr16, r16, retaining only the lower byte in R23 and clearing R24, as higher-order precision is not required (kines 12–14). Next, operand *B* is squared using mulr17, r17, and its result is accumulated with $A^{2}$ through add and adc instructions to form A2+B2 (lines 17–19). The final arithmetic step subtracts this squared sum from the previously computed CD using sub and sbc, with the resulting low byte stored in R20 (lines 22–24). Upon completion, execution jumps directly to the end of the program, bypassing the remaining base code (line 26).

**Listing 4:** Update C’s assembly code. Checks if to calculate *Z* = (*C* × *D*) − (*A*^2^ × *B*^2^) after a point in the base program’s code.

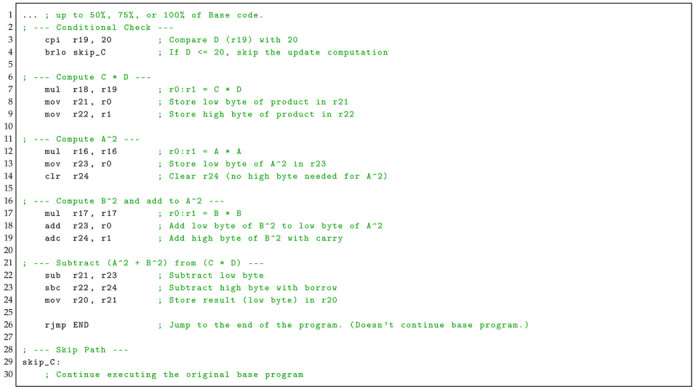



To increase the number of program variations, each update is inserted at three distinct points within the Base program’s instruction sequence: approximately 50%, 75%, and after 100% of the original Base program’s execution. By applying each update at these injection points, we generate a total of nine unique program variations, each representing a different combination of original and modified execution sequences.

In our experiments, we collect 2500 samples for each update program for evaluating purposes. To obtain anomalous cases, we also capture 2500 samples where a subtle add instruction injection is introduced into each program variation at roughly the 45% point. These anomalous EM signals exhibit only minor deviations in their waveform morphology compared with their benign counterparts, making accurate differentiation particularly challenging. This setup allows us to rigorously assess the model’s ability to detect subtle, instruction-level modifications in execution traces.

**Figure 7:**
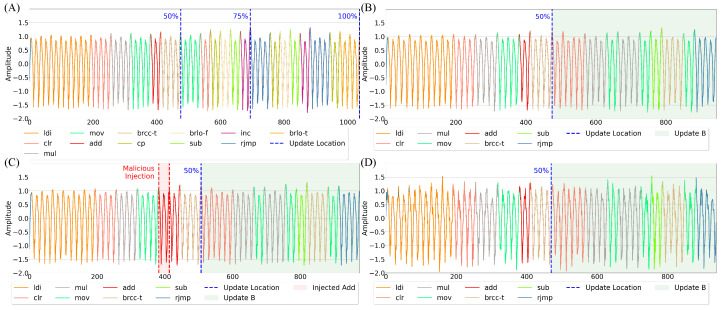
EM sample with instructions indicated of (**A**) the Base program, (**B**) update 50 B, (**C**) update 50 B with malicious injection of an add instruction, and (**D**) update 50 B at an SNR of 15.

To analyze the impact of real-world noise on EM-based anomaly detection, different levels of White Gaussian Noise (WGN) are artificially introduced into the captured signals for both benign and anomalous cases, generating multiple datasets. This controlled noise injection allows for systematic evaluation under varying noise conditions while maintaining a measurable Signal-to-Noise Ratio (SNR). Example of emissions inside the dataset are given in Figure 7.

### 5.4. Training Programs

Ideally, the GAN model would be trained on a comprehensive library containing all possible—or at least the most frequently observed—ESDs and their corresponding EM signal patterns. Each ESD entry in this library is associated with multiple EM signal samples to capture intra-state variability and ensure accurate representation of the underlying emission modality. Such a dataset provides near-complete coverage of potential program states, enabling the generative model to learn both common and subtle variations in EM behavior across instruction executions.

In practice, however, the update programs used in our experiments involve only a specific subset of ESDs. Therefore, we constructed a minimized yet representative dataset by extracting the relevant ESDs and corresponding EM signals from four distinct toy training programs, each captured 2500 times. Collectively, these programs cover every ESD observed in the finite update programs when considering instruction sequences ranging from one to four operations, which would typically be expected in a comprehensive library. To illustrate this coverage, a segment of one training program is shown below.

In Listing 5, the sequence mov–mul–mov–clr (lines 2–5) appears in update C, while clr–clr–clr–mul (lines 11–14) is found in update A.

**Listing 5:** Example of a segment code from a Train Program’s assembly code.

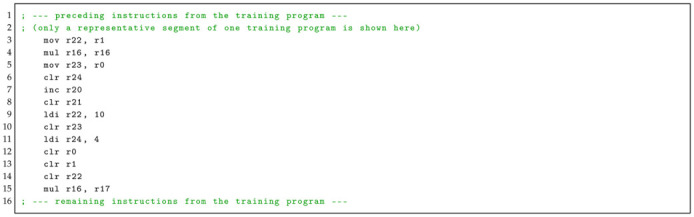



To further analyze how ESDs influence EM signal fidelity, the training programs incorporate multiple variations where preceding instructions, operands, and register values differ for the same ESD observed in the update programs. This variation allows the model to learn not only direct instruction-to-signal mappings but also the contextual dependencies arising from different execution histories. By capturing this diversity, the GAN generalizes across a wide range of execution patterns, enabling robust EM signal synthesis even for previously unseen yet structurally similar instruction sequences.

### 5.5. Preprocessing

No preprocessing was applied to the signals before training the GAN model or generating EM emissions. However, because EM emissions are amplitude-modulated, the peak values serve as key features that indicate the executed instruction at any given time. Notably, within each cycle (comprising two waves), only the first peak (major peak) exhibits significant variation between different instructions, while the second peak remains relatively static. As a result, our approach prioritizes these major peaks for fidelity assessment and anomaly detection phases only.

**Figure 8:**
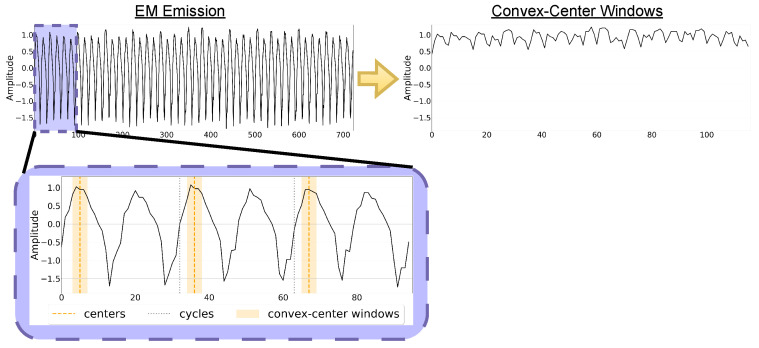
Convex-center windows.

Moreover, since different instructions can share the same major peak amplitude but differ in the overall signal shape, we refine our analysis by focusing on the center of the convex curve surrounding the major peak. To capture variations in signal morphology, we extract a localized set of time-indexed samples around this center or *convex-centered window* (CCW), as illustrated in Figure 8. This approach ensures that both the amplitude and shape of the major peak are preserved, enabling finer distinction between instruction patterns and improving anomaly detection. In addition, by realigning traces around a stable morphological feature, the CCW method mitigates the effects of clock drift—minor timing deviations between captures caused by oscillator instability or sampling rate mismatch—without requiring computationally intensive alignment techniques such as dynamic time warping.

## 6. Experimental Evaluation

The following section presents the experimental results, assessing the effectiveness of the proposed framework in generating high-fidelity EM signals and evaluating the feasibility of using synthetic signals to train SCAD models for detecting subtle modifications in a program’s base ASM code.

### 6.1. Synthetic EM Fidelity

To assess how effectively the proposed framework generates EM signals that replicate those captured from the target AVR CPU, we conduct evaluations at two levels of granularity: full-signal and cycle-level analyses. The full-signal evaluation provides a global measure of similarity between synthetic and real EM traces, offering an overall view of waveform alignment. This analysis serves three key purposes: (1) establish the achievable fidelity relative to real emissions; (2) determine the contribution of ESD conditioning—specifically, state information (i.e., inclusion of operands and register values) compared to using instruction-only inputs; and (3) identify which GAN architecture yields higher-fidelity synthetic signals. In contrast, the cycle-level evaluation focuses on localized waveform morphology, examining fine-grained consistency across individual instruction cycles using the best-performing model identified from the full-signal analysis.

#### 6.1.1. Full-Signal Fidelity Evaluation

In the full-signal evaluation, we assess how accurately the generated signals replicate the training data by comparing them to the real signals used to construct the training library. The primary goal of this experiment is to determine whether the generative models can reproduce entire waveform structures with sufficient fidelity to serve as substitutes for physically captured EM traces. This allows us to evaluate the generative framework’s ability to generalize beyond isolated instruction-level synthesis and capture the broader temporal and morphological characteristics of real emissions.

**Figure 9:**
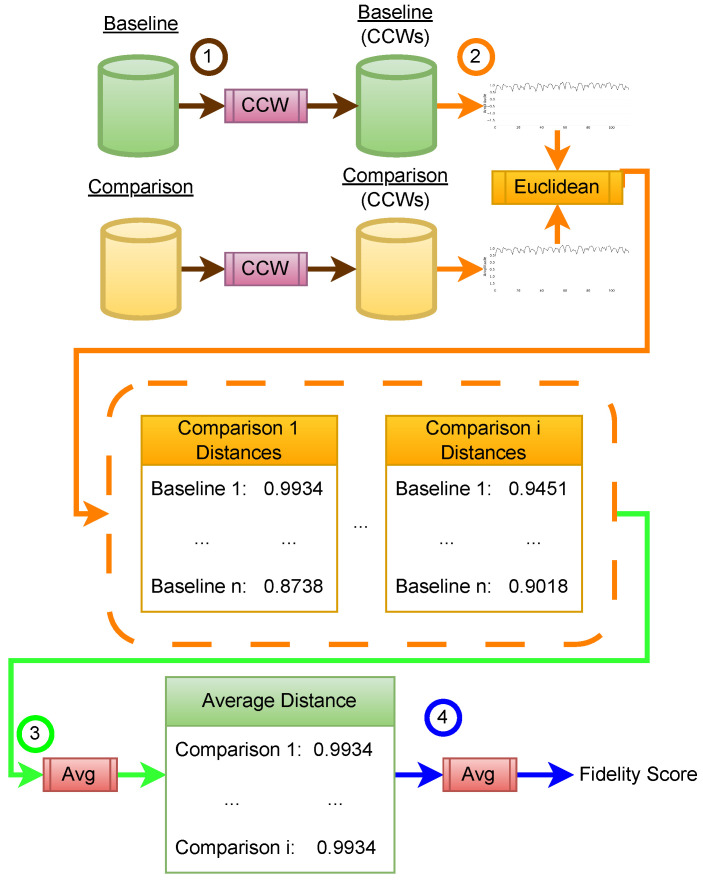
Method for calculating the fidelity between two datasets: ① extract CCWs; ② compute Euclidean distances between all baseline–comparison; ③ average distances per comparison signal; and ④ average again to obtain fidelity score.

For each training program, we collect 150 synthetic signals and compare them against 150 real captured signals, which serve as the baseline. The comparison is based on the Euclidean distance between convex-centered windows (CCWs) and is illustrated in Figure 9. Additionally, the algorithm used for this evaluation is provided in Appendix A.

The method begins by extracting CCWs from both the baseline and comparison datasets ①. For each comparison signal, its Euclidean distance to all baseline signals is calculated ② and averaged ③, producing a list of per-signal distance values that is then averaged again ④ to yield a single fidelity score. This process is repeated for each training program, and the individual fidelity scores are summed to obtain the total fidelity.

While Euclidean distance is a common similarity metric in signal analysis, we acknowledge that in traditional side-channel settings, it can be highly sensitive to trace misalignment (jitter), amplitude scaling, and localized temporal distortions. Several alternative measures exist, including Dynamic Time Warping (DTW) [36] for elastic temporal alignment, correlation-based metrics [37], or distributional divergences such as Wasserstein distance [38] for comparing statistical trace distributions. However, in our experimental setup, two design choices substantially mitigate the typical weaknesses of Euclidean metrics. First, all signals are normalized before comparison, ensuring that the metric is insensitive to uniform amplitude scaling between captures. Second, rather than evaluating full-length traces, the comparison is performed exclusively on CCWs, which isolate the stable, instruction-dependent peak morphology and eliminate most of the temporal jitter present in raw EM signals. Note, as illustrated in Figure 8, this process is done per cycle and removes the curse of dimensionality. Because these CCWs are extracted relative to a consistent morphological anchor, their temporal alignment is effectively preserved, making Euclidean distance a robust and interpretable measure in this constrained setting.

To establish a benchmark for similarity, we first measure the fidelity of real captured signals by comparing a base reference set of EM traces against additional real signals obtained from the same training programs. This represents the ideal case and defines the highest achievable similarity for evaluating synthetic quality. Building upon this benchmark, we then assess the fidelity of our framework by comparing synthetic EM signals generated by both the ResGAN and the proposed 1DCNNGAN models against the real baseline signals captured from the target device. In principle, it is reasonable to expect that the synthetic EM emissions with enough ESD information would closely mirror their real counterparts—both in structure and spectral morphology—yielding fidelity scores nearly indistinguishable from those of real captured signals. However, the CWGAN-GP model for code-to-EM generation may not need computationally heavy Resnet-50 layers to accomplish high fidelity.

To comprehensively evaluate signal fidelity, the models were trained and tested using instruction sequence lengths ranging from one to four instructions (1 Inst., …, 4 Inst. in Table 3). This variation allows us to investigate how contextual depth—i.e., including preceding instructions—affects the model’s ability to reproduce realistic EM morphology. Because each instruction’s emission is influenced by its prior operations due to residual charge effects and pipeline overlap, longer instruction sequences capture these dependencies more effectively, yielding higher waveform similarity to real signals.

Furthermore, to analyze the contribution of state information to generative fidelity, we evaluate two ESD configurations denoted by I and I/O/RV, where I represents instruction-only input, O includes operand values, and RV incorporates register values prior to execution. These descriptors capture progressively richer contextual information about the device’s internal state.

**Results:** The quantitative results of the full-signal fidelity evaluation are summarized in Table 3. The Euclidean distance between real captured signals and their baseline counterparts is 8.745, establishing the reference or best-case scenario that represents the highest achievable similarity using real data alone. When analyzing synthetic signals generated without state information—trained solely on instruction sequences—both models show gradual improvement as additional instruction context is provided. For ResGAN, the distance decreases from 13.882 when trained on single instructions to 12.164 when using four-instruction inputs. The 1DCNNGAN architecture, however, exhibits instability with limited input, producing a large distance of 248.967 for one instruction but converging rapidly to 11.772 once four instructions are considered.

Incorporating ESDs with state information—specifically operands and register values—further enhances fidelity across all sequence lengths, reducing the seen Euclidean distance by roughly 13%. *This reveals the key trend that adding operand and register-value conditioning (I/O/RV) consistently lowers Euclidean distance for both models, improving the similarity between synthetic and real EM signals.* The best performance is achieved using four instructions with full I/O/RV conditioning, yielding distances of 10.216 for ResGAN and 10.203 for 1DCNNGAN—both approaching the real-signal reference.

**Conclusions:** While ResGAN demonstrates more consistent behavior across reduced-context inputs—those trained on limited instruction information—owing to its deeper residual architecture and inherent gradient stability, the proposed 1DCNNGAN ultimately surpasses it when more contextual information is available. At these higher-context levels, meaning when the model is trained on sequences of multiple consecutive instructions (e.g., three–four instructions), 1DCNNGAN achieves slightly lower Euclidean distances compared with ResGAN. This improvement arises because longer instruction sequences allow the model to capture inter-instruction dependencies.

Moreover, when comparing input configurations, the inclusion of O and RV information alongside instruction data consistently improves fidelity for both models. This demonstrates that state-aware conditioning enables the generator to account for variations in the device’s internal electrical and logical states, producing more realistic waveform structures. Collectively, these results show that while ResGAN benefits from its residual depth under limited input conditions, the more lightweight 1DCNNGAN achieves equal or superior fidelity once sufficient contextual and state information is incorporated—indicating that the heavy ResNet-50 backbone is not necessary for high-quality EM signal synthesis.

#### 6.1.2. Cycle-Level Fidelity Evaluation

**Figure 10:**
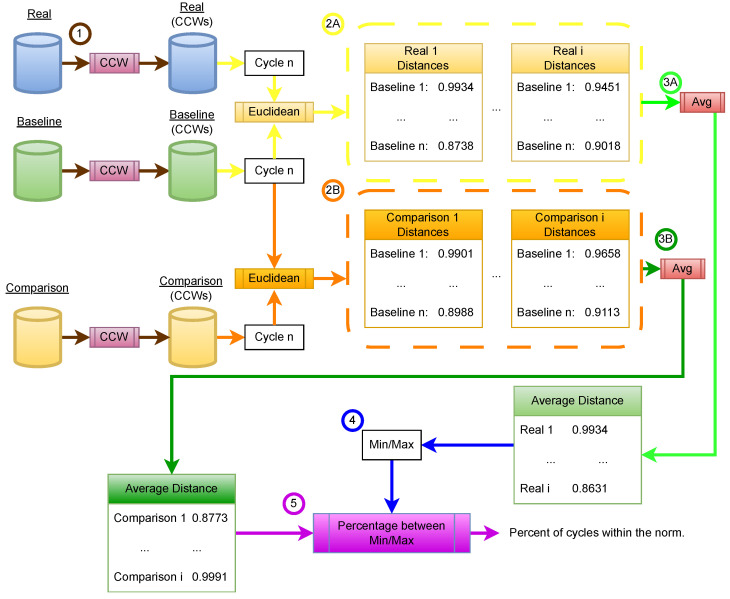
Method for calculating the percent of cycles that fall within the expected normal distribution.

In the second evaluation, we assess the fidelity of the best-performing GM by comparing individual instruction cycles using a defined normalcy range derived from real EM signals, as shown in Figure 10. CCWs are extracted from baseline, real, and synthetic datasets ①, and cycle-specific distances are computed between each comparison sample and the baseline, mirroring the earlier fidelity approach. These distances are used to establish a normal range based on real-to-baseline comparisons ④, against which synthetic-to-baseline distances are evaluated ⑤. The final metric reflects the percentage of synthetic cycles across all update programs that fall within this normal range, using datasets of 800 samples each.

**Figure 11:**
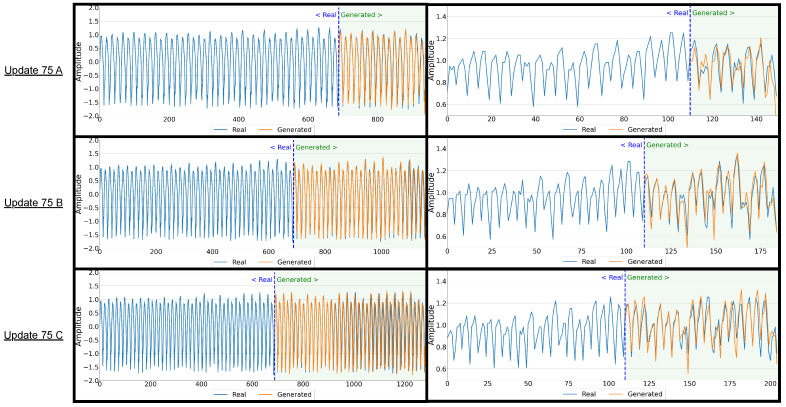
Sample comparisons between the real- and generated-signal segments for the full emission (**left**) and CCWs (**right**).

**Results:** Across the nine update programs evaluated, the comparison between real captured signals and generated signals reveals generally strong fidelity of the synthetic data. For each update program—specifically, updates occurring at 50%, 75%, and 100% execution points for programs A, B, and C—the overlay graphs given in Figure 11 show that the generated signals closely track the real captured signals when viewed over the CCW regions. When analyzing the segments after the dark blue line (generated-to-real comparisons) demonstrate slight variance but overall maintain a similar structure, suggesting that the generative model successfully captures the key signal dynamics across varying program states.

**Figure 12:**
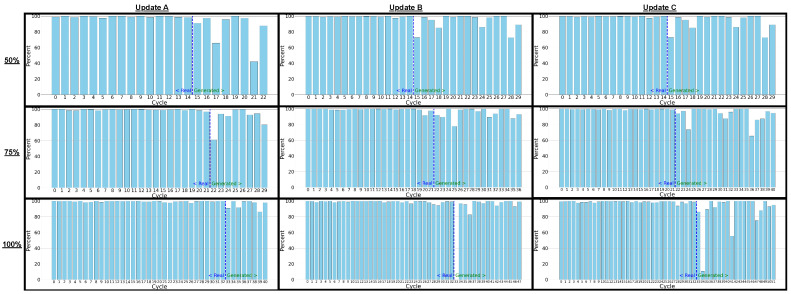
For each update type (A, B, and C) and insertion point within the Base program (50%, 75%, and 100%), the bars report the percentage of instruction cycles whose CCW distance falls within the norm. In each graph, the left of the dark blue line shows repeat-capture consistency (real to real), and the right of the line shows generative fidelity (generated to real) for the updated code sequences.

The bar graph analyses—representing the percentage of generated CCWs falling within the normal real captured range—provide a more quantitative measure of fidelity, as seen in Figure 12. When comparing real-to-real signal variants, performance across the nine update programs remains consistently high, averaging about 95–97%, confirming the stability of genuine EM captures under repeated acquisitions. In the real-to-generated comparisons, roughly 8–18% of instruction cycles fell outside the defined normal range, yielding average fidelity scores of 82–92% across most update cases. The few failed cycles were predominantly concentrated in transitions immediately surrounding the inserted update blocks—particularly at the injection points of update C, where conditional branching and register reuse introduced nonlinear state transitions that the model reproduced less precisely. A smaller number of deviations also appeared in the first few cycles following update regions in updates A and B, likely due to transient charge redistribution and operand-dependent amplitude shifts not fully captured by the conditioning descriptors. However, some programs, particularly at the 75% and 100% update points for programs A and B, achieve performance percentages close to real-to-real comparisons, highlighting that the generative model can reproduce emission behavior with high reliability even under significant program changes.

**Conclusions:** The strong overlay alignment observed in Figure 11 and the high CCW success rates summarized in Figure 12 together confirm that the proposed 1DCNNGAN model is capable of reproducing realistic, instruction-level EM emissions with minimal deviation from real captured behavior. Although the generated-to-real fidelity is marginally lower than the real-to-real baseline—typically by only 5–10 percentage points—the vast majority of cycles remain within the normal range of real emissions. This indicates that the synthetic signals preserve nearly the same temporal coherence and amplitude morphology as genuine captures.

Importantly, the slight reduction in fidelity is localized rather than systematic: most deviations arise in the few cycles immediately after the inserted update regions, where abrupt context transitions, branch instructions, and register re-initialization create short-lived nonlinearities that are difficult to model perfectly. Beyond these transition zones, the generated emissions maintain near-identical morphology to real signals, as reflected in the per-cycle bar distributions, where nearly every update variant achieves results closely clustering around the 85–95% range.

Furthermore, the use of real captured emissions up to the point of each update substantially contributes to the near-perfect fidelity across the whole signal. By anchoring the generated segment to authentic pre-update waveforms, the framework avoids compounding generative uncertainty across the full signal and confines potential artifacts only to the synthesized region. This hybrid strategy ensures that high-fidelity reproduction is retained in all unmodified sections while allowing flexible adaptation to code changes.

### 6.2. Model Efficiency

In the following section, we evaluate the efficiency of the two GAN models by examining two key characteristics: the average time required to train each epoch across the various ESD configurations, and the memory footprint of each model. Together, these metrics provide insights into the practical applicability of each approach for real-world deployment. Based on the computational cost of ResNet-50, we expect 1DCNNGAN to be computationally less expensive than ResGAN and thus faster to train.

#### 6.2.1. Memory Footprint

To assess the practical efficiency of each model in real-world scenarios, we evaluate their memory footprint across the different ESD configurations. Memory usage is a critical factor when deploying models on resource-constrained systems, as it directly impacts the feasibility of real-time execution, scalability across multiple devices, and integration into embedded or edge computing environments. Models with lower memory requirements are more adaptable to a wider range of deployment setups, enabling faster inference, reduced hardware costs, and greater overall system stability. For this evaluation, we assume that the model takes 4 bytes per parameter and evaluate with a batch size of 32.

**Results:** Table 4 presents the total memory usage of both models across the different ESD configurations. Owing to its deep architecture based on the ResNet-50 backbone, ResGAN exhibits a substantially higher memory footprint—approximately 99.8 MB across all configurations. In contrast, 1DCNNGAN, which employs a lightweight design composed primarily of a few 1D CNN and dense layers, requires only a fraction of that memory. Its largest configuration, trained with full I/O/RV conditioning and four-instruction sequences, consumes merely 9.217 MB.

#### 6.2.2. Time to Train

For the time-to-train experiment, each model was trained on an identical dataset and batch size corresponding to the same ESD instance to ensure a consistent evaluation. Additionally, both models were trained using the same NVIDIA RTX 4090 GPU to maintain a fair hardware baseline. Evaluating training time is critical in real-world scenarios, as faster training reduces development overhead, lowers computational costs, and facilitates quicker adaptation.

**Results:** The results of the time-to-train evaluation are summarized in Table 5. The ResGAN model demonstrates high efficiency when trained on limited input data, completing epochs in approximately 3 s and 12 s for one- and two-instruction configurations, respectively. However, training time increases sharply with larger input sequences and additional state information, reaching up to 254 s per epoch when trained with full I/O/RV conditioning and four-instruction sequences. In contrast, the proposed 1DCNNGAN maintains consistently lower training times across configurations with more input, requiring at most 57 s per epoch under the same I/O/RV and four-instruction setup.

**Conclusions:** Based on the training time and memory footprint evaluations, 1DCNNGAN is substantially more computationally efficient than ResGAN. When considering the best-fidelity configuration identified in Section 6.1 (the 4Inst.I/O/RV setup), both models achieved near-equivalent signal fidelity. Despite this near-identical fidelity, 1DCNNGAN completed training in only 57 s per epoch compared with 254 s for ResGAN and required merely 9.2 MB of memory versus 99.9 MB. This roughly represents a 4.5× reduction in training time and over a 10× reduction in memory usage.

### 6.3. Anomaly Detection

**Figure 13:**
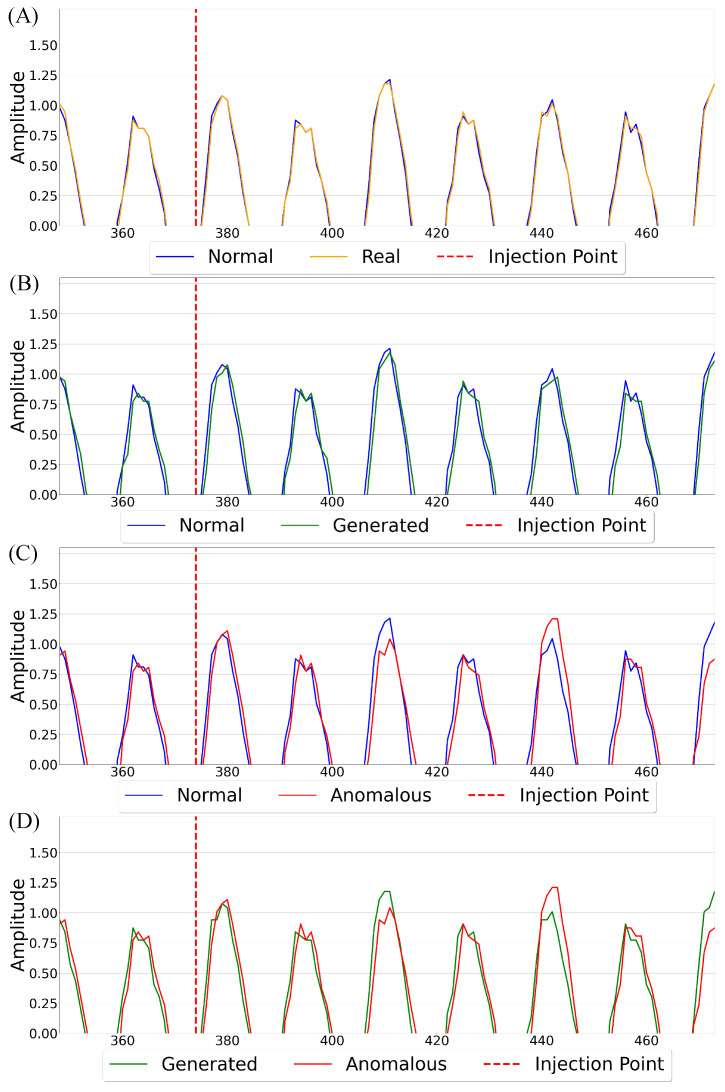
Comparison of (**A**) real normal–real normal, (**B**) synthetic normal–real normal, (**C**) real normal–real anomalous, and (**D**) synthetic normal–real anomalous traces around the injected anomaly.

This section evaluates the effectiveness of the proposed framework for anomaly detection when the models were trained on synthetic EM signals. The goal of this experiment is not to develop a new detection technique but rather to assess whether synthetic EM emissions, generated from ESDs and the proposed GAN model, can effectively replace real EM data for training anomaly detection models—particularly in program updates scenarios where re-fingerprinting would typically be required. Thus, the distinction between the real and synthetic signals should be minimal, while the differences between normal and anomalous signals should be high, as illustrated in Figure 13.

**Figure 14:**
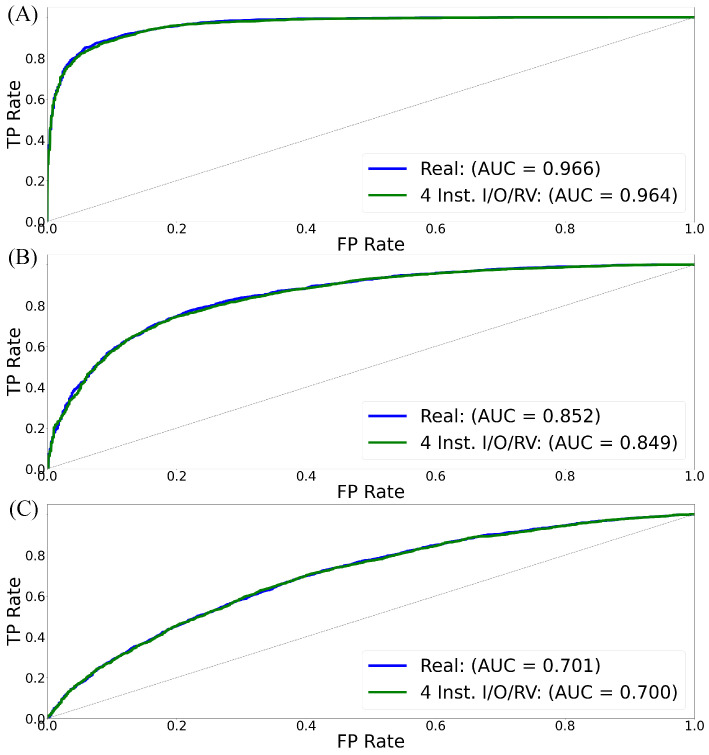
Comparison of AUC results for update 75 A at (**A**) an SNR of 20 (**B**) an SNR of 15, and (**C**) an SNR of 10.

To this end, we conduct two core experiments. In the first, we train a semi-supervised kNN anomaly detection model on real EM signals, establishing a baseline. In the second, the same model is trained on synthetic EM signals. These were generated by 1DCNNGAN, which was in turn trained only on sequences of four instructions. Next, we analyze the benefit of added ESD information by retraining 1DCNNGAN with additional operand and register information. Furthermore, we evaluate the effectiveness of 1DCNNGAN compared with ResGAN by repeating the evaluation with ResGAN on the ESDs with operand and register information. For all experiments, 5-fold cross-validation is used with 2000 training samples and 1000 test samples (500 benign and 500 anomalous) per fold. All tests are repeated across nine program variants and are evaluated under varying Signal-to-Noise Ratios (SNRs) of 20, 15, and 10 dB to reflect realistic environmental noise. Noise was intentionally introduced and evaluated to emulate the electromagnetic interference and operational variability that naturally occur in real-world capture environments, ensuring that the results reflect deployment-level robustness rather than ideal laboratory conditions. Detection performance is measured using Area Under the Curve (AUC) of the Receiver Operating Characteristic (ROC), accuracy (ACC), F1 score, precision (PREC), and recall (REC). While advanced denoising methods (e.g., Principal Component Analysis (PCA) and autoencoders) could improve performance under high noise, our focus is on evaluating the fidelity of synthetic signals in comparison to real ones. Figure 14 illustrates the overall trend in the AUC when comparing the use of real emissions to train on and synthetic emissions produced by 1DCNNGAN trained on four instructions and state information. Overall results are provided in Table 6.

#### 6.3.1. kNN and Real EM Signals

To establish the performance baseline, we train the anomaly detection model using real EM signals with no synthetic components. This represents the ideal case, where the detector is trained on ground-truth EM emissions.

**Results:** When trained on real EM signals, the kNN model consistently demonstrates high detection performance across all programs under low-noise conditions. At SNR = 20, AUC scores exceed 0.96 for most program variants, with several—such as 75 B, 100 B, and 100 C—reaching values above 0.99. However, the effects of noise become more pronounced at SNR = 10, where AUC scores for several smaller or earlier-injected updates, such as 50 A and 75 C, fall into the range of 0.63–0.76. This reduction is particularly evident in precision and recall values, showing that model confidence and sensitivity degrade with higher noise levels. Updates involving longer instruction sequences, like 100 B and 100 C, retain better performance at lower SNRs.

**Conclusions:** Even a simple kNN model performs exceptionally well in low-noise environments when trained on real EM signals. However, its detection capability degrades as noise increases, emphasizing the inherent challenges in signal-based anomaly detection under realistic conditions.

#### 6.3.2. kNN and Synthetic EM Signals (ESD: 4 Instruction Sequence and State)

In the next stage of our evaluation, we investigate the effects of ESDs. The semi-supervised kNN model is retrained using synthetic signals where the updated regions are generated by a state-aware GM. We hypothesize based on the previous fidelity experiments that the inclusion of ESD conditioning—comprising instruction, operand, and register-value information—will improve anomaly detection performance by enabling the generative model to capture finer variations in state transitions and amplitude morphology that correspond to real execution behavior. Consequently, we expect that anomaly detection models trained on ESD-conditioned synthetic signals will yield higher accuracy and stability across varying update programs and noise levels, narrowing the gap between real- and synthetic-trained detectors. The outcomes of this experiment are given in full detail in the middle of Table 6.

**Results:** Incorporating operand and register-value information into the ESDs leads to measurable, context-dependent improvements in anomaly detection accuracy across nearly all update programs and noise levels. When comparing synthetic EM signals generated from a model trained only on four-instruction sequences (4Inst.I) with those produced by a state-aware model including operands and register values (4Inst.I/O/RV), Table 6 shows consistent improvements in AUC, F1, and recall performance.

Under high-quality conditions (SNR = 20 dB), the instruction-only models typically fall by 0.03–0.05 AUC below the real-signal baseline, while the ESD-enhanced models reduce this difference to 0.01–0.02, with several cases (50 B, 75 C, and 100 B) matching or slightly above the real-trained performance. As the SNR decreases, the gap widens for instruction-only synthesis—dropping by 0.04–0.06 AUC on average at 15 dB and by up to 0.10 AUC at 10 dB—whereas the I/O/RV-conditioned models recover roughly half of that loss (e.g., 75 A improves from 0.8217 to 0.8485 at 15 dB and from 0.6820 to 0.6996 at 10 dB). These gains persist across all supporting metrics, yielding 2–3 percentage-point improvements in F1 and recall under noisy conditions. The overall pattern indicates that state-aware conditioning enables the generator to better preserve temporal and amplitude characteristics critical to the kNN decision boundary, particularly as environmental noise increases.

**Conclusions:** The findings confirm that state-aware conditioning is essential to achieving near-real anomaly detection performance using synthetic EM signals. While instruction-only synthesis captures general waveform structure, it lacks the fine-grained variability in amplitude and timing introduced by state conditions needed for stable detection, especially under noise. Embedding operands and register values within the ESDs allows the GM to retain this context, closing the performance gap to within roughly 1–2 % AUC of real-signal models at 20 dB and maintaining resilience even at 10 dB. Thus, ESD-conditioned synthesis not only validates our previous hypothesis but also establishes a practical baseline for post-update retraining without re-fingerprinting.

#### 6.3.3. kNN and Synthetic EM Signals (1DCNNGAN vs. ResGAN)

The final evaluation compares the anomaly detection performance when synthetic EM emissions generated by either the ResGAN or 1DCNNGAN model are used to train the semi-supervised kNN detector. In this analysis, the results obtained from 1DCNNGAN—trained on four-instruction sequences with operand and register values (as discussed previously)—are directly compared with those of ResGAN trained under the same ESDs. The corresponding ResGAN results are reported in the far-right column of Table 6. Given that both models achieved comparable fidelity in Section 6.1, it is theoretically expected that their anomaly detection performance will be closely aligned, with 1DCNNGAN potentially exhibiting a modest advantage.

**Results:** The table reports the AUC performance of a kNN anomaly detection model trained solely on synthetic EM signals produced by each GAN under the same configuration—four-instruction ESDs incorporating instruction, operand, and register-value information (4Inst.I/O/RV). Across all nine updated program variants, the ResGAN-based detector maintains strong discriminative ability even in the presence of noise. At the highest signal quality (SNR = 20), AUC values range from 0.9228 (50 A) to 0.9989 (75 B and 100 B), showing excellent separation between benign and anomalous traces using only synthetic training data. When the SNR decreases to 15, performance remains robust, with most AUC scores between 0.85 and 0.95, while at SNR = 10, the detector still sustains AUC values above 0.67 for all cases—demonstrating that ResGAN-based synthetic signals can effectively support anomaly detection even under degraded conditions.

**Conclusions:** A direct comparison between 1DCNNGAN and ResGAN indicates that both generative models achieve nearly identical signal fidelity and detection performance. Across the various program variants and noise levels, their AUC values differ only marginally (mostly within ±1), suggesting comparable effectiveness in enabling SCAD through synthetic training data.

## 7. Discussion and Future Directions

The results presented in this work demonstrate the strong potential of GMs to synthetically create EM side-channels given a program’s ASM code, particularly when updates to software occur. Thus, the proposed framework eliminates the need for re-fingerprinting when a benign software modification (e.g., software update) occurs. In these experiments, we introduce modified/updated execution paths and use the GM to synthesize the corresponding EM emissions offline. Through comparative analysis across alternative program updates and execution points, the generated signals showed similarity to real captured emissions. Overlay comparisons confirmed that synthetic signals closely tracked the morphological characteristics of authentic EM waveforms. CCW analyses further validated that critical waveform regions remained within the natural variability bounds observed across real captures. Quantitatively, the synthetic signals consistently achieved high match percentages for the CCWs—averaging between 82% and 92%—compared with real-to-real comparisons, which averaged only 95% due to naturally occurring signal variants. These findings suggest that GM can substantially alleviate the burden of re-fingerprinting, offering a scalable path toward maintaining SCAD systems through frequent software updates.

Further supporting this conclusion, anomaly detection results demonstrated that models trained on synthetic signals generated using the four-instruction sequences with the operand and register-value ESD-conditioned GM achieved classification performance closely matching that of models trained on real EM captures. Specifically, across all update scenarios, the real EM-trained anomaly detection models achieved AUCs ranging from 0.96 to 0.98, while the models trained on synthetic signals achieved AUCs between 0.95 and 0.97. Importantly, this strong performance persisted even under varying SNR conditions, including degradations at SNR levels of 20 dB, 15 dB, and 10 dB. Furthermore, while comparable fidelity and anomaly detection results can be achieved using the prior state-of-the-art ResGAN model, the proposed 1DCNNGAN demonstrates superior efficiency—requiring only about one-tenth of the memory and training in roughly one-fifth of the time.

While this study demonstrates the potential of synthetic EM signal generation, it remains preliminary, and several challenges must still be addressed before the approach can be broadly generalized. Future work should focus on further improving the robustness and adaptability of generative models for EM signals. Towards this end, we aim to experiment with transformer-based architectures and state space models. Additionally, scaling the approach to cover a broader range of architectures and instruction sets without retraining from scratch remains an open challenge. Investigating domain adaptation, transfer learning, and hybrid conditioning methods could enable generative models to generalize across diverse platforms, ensuring wide applicability in operational environments. Furthermore, future efforts will extend beyond assembly code to include representations such as hexadecimal and binary formats while expanding the GAN framework to train on full-length EM traces rather than being limited to instruction-level cycle libraries.

An important requirement for real-world applicability lies in addressing nonlinear program execution, where hardware interrupts, preemption events, and context switches introduce asynchronous signatures that deviate from strictly linear instruction flows. While the present work focuses on linear sequences, future research will investigate how such events alter EM morphology and whether generative models can be conditioned to recognize and reproduce these characteristic interrupt signatures. This effort will be supported by extending our methodology to additional architectures besides AVR, such as ARM, MIPS, and RISC-V, enabling controlled evaluation of interrupt-driven behavior and more complex execution pipelines.

Beyond architectural considerations, a significant challenge arises from physical acquisition variability, including changes in probe positioning, sensor-to-target distance, board-level localization, and device-to-device manufacturing differences. These factors introduce measurable shifts in EM trace distributions that can degrade generative model fidelity. To address this, we plan to explore transfer learning and domain adaptation strategies capable of compensating for such measurement-induced variability, thereby enabling generative models to remain robust under practical deployment conditions.

To further validate and strengthen the proposed approach, future work will also expand the scope of generative and detection methods evaluated. Beyond CWGAN-GP, we plan to examine additional generative architectures—such as diffusion models and transformer-based sequence generators—that have demonstrated strong performance in the text-to-speech synthesis domain. On the anomaly detection side, we will assess a broader set of models to determine whether synthetic EM traces preserve discriminative structure across diverse detection paradigms. Finally, we aim to evaluate the system under a wider range of threats, including other code-modification attacks and control-logic bombs, to better understand the framework’s robustness against realistic adversarial scenarios.

## Figures and Tables

**Table 1:** List of acronyms.

Acronym	Definition
ACC	Accuracy
AUC	Area Under the Curve
AVR	Alf-Egil Bogen and Vegard Wollan RISC architecture (Microchip)
CCW	Convex-centered window
CNN	Convolutional Neural Network
CPU	Central Processing Unit
CWGAN-GP	Conditional Wasserstein GAN with Gradient Penalty
dB	Decibels
DTW	Dynamic Time Warping
EM	Electromagnetic
ESD	Execution State Descriptor
GAN	Generative Adversarial Network
GM	Generative model
I	Instructions
MB	Megabits
MCU	Microcontroller unit
O	Operator
PCA	Principal Component Analysis
PREC	Precision
REC	Recall
ROC	Receiver Operating Characteristic
RV	Register value
SCAD	Side-Channel-based Anomaly Detection
SNR	Signal-to-Noise Ratio
WGN	White Gaussian Noise

**Table 2:** Comparison of generative models for EM synthesis. “Partial” indicates that a model can satisfy the criterion only under specific architectural modifications, substantial computational scaling, or restrictive assumptions.

Model	Parallel	Efficient	Low-Data	Stable	Conditional	High Fidelity
Autoregressive	No	No	Partial	Yes	Yes	Yes
Diffusion	No	No	Partial	Yes	Yes	Yes
Transformer	Partial	No	Partial	Partial	Yes	Yes
VAE	Yes	Yes	Yes	Yes	Partial	No
CWGAN-GP	Yes	Yes	Yes	Yes	Yes	Yes

**Table 3:** Euclidean distance between baseline real EM signals and real or synthetic variants under different input configurations (I: instruction; O: operand; RV: registers) and sequence lengths.

	ResGAN				1DCNNGAN				Real
	**1 Inst.**	**2 Inst.**	**3 Inst.**	**4 Inst.**	**1 Inst.**	**2 Inst.**	**3 Inst.**	**4 Inst.**	**N/A**
**I**	13.882	12.459	12.354	12.164	248.967	25.560	12.103	11.772	8.745
**I/O/RV**	11.325	11.031	10.989	10.216	10.853	11.929	11.730	10.203	8.745

**Table 4:** Total memory usage for each model given in megabytes (MB).

	ResGAN				1DCNNGAN			
	**1 Inst.**	**2 Inst.**	**3 Inst.**	**4 Inst.**	**1 Inst.**	**2 Inst.**	**3 Inst.**	**4 Inst.**
**I**	99.812	99.814	99.816	99.818	3.760	3.887	4.014	4.141
**I/O/RV**	99.877	99.883	99.889	99.894	8.075	8.456	8.836	9.217

**Table 5:** Approximate time in seconds to train the GMs for one epoch under each of the following ESD conditions when utilizing an NVIDIA RTX 4090 GPU.

	ResGAN				1DCNNGAN			
	**1 Inst.**	**2 Inst.**	**3 Inst.**	**4 Inst.**	**1 Inst.**	**2 Inst.**	**3 Inst.**	**4 Inst.**
**I**	3	12	22	34	15	16	17	23
**I/O/RV**	78	240	246	254	51	54	56	57

**Table 6:** Anomaly detection results. Highlighted cells indicate key comparative regions: red = heavy degradation in instruction-only models, green = strong recovery under noise due to ESD conditioning, and blue = synthetic performance equivalent to real signals.

		Real			1DCNNGAN						ResGAN		
		**N/A**			**Synth EMs. 4 Inst. I**			**Synth EMs. 4 Inst. I/O/RV**			**Synth EMs. 4 Inst. I/O/RV**		
**Program**	**Metric**	**SNR**			**SNR**			**SNR**			**SNR**		
		**20**	**15**	**10**	**20**	**15**	**10**	**20**	**15**	**10**	**20**	**15**	**10**
**50 A**	**AUC**	0.8713	0.7385	0.6380	0.9263	0.7897	0.6715	0.9284	0.7932	0.6773	0.9228	0.7976	0.6856
	**ACC**	0.7950	0.6872	0.6058	0.8510	0.7248	0.6294	0.8532	0.7296	0.6378	0.8490	0.7330	0.6424
	**F1**	0.8026	0.7167	0.6776	0.8528	0.7450	0.6865	0.8547	0.7473	0.6878	0.8510	0.7518	0.6947
	**PREC**	0.7939	0.6691	0.6143	0.8679	0.7220	0.6372	0.8603	0.7236	0.6574	0.8494	0.7229	0.6561
	**REC**	0.8144	0.7516	0.5760	0.8472	0.7568	0.6292	0.8488	0.7664	0.6092	0.8528	0.7644	0.6632
**50 B**	**AUC**	0.9961	0.9293	0.7757	0.9944	0.9190	0.7656	0.9970	0.9330	0.7809	0.9947	0.9299	0.7842
	**ACC**	0.9706	0.8548	0.7364	0.9660	0.8440	0.7072	0.9756	0.8610	0.7182	0.9676	0.8604	0.7172
	**F1**	0.9706	0.8556	0.7364	0.9663	0.8465	0.7295	0.9756	0.8623	0.7369	0.9679	0.8605	0.7385
	**PREC**	0.9723	0.8684	0.7152	0.9642	0.8388	0.7121	0.9757	0.8863	0.7300	0.9635	0.8662	0.7259
	**REC**	0.9716	0.8516	0.7332	0.9740	0.8592	0.7012	0.9764	0.8584	0.7200	0.9768	0.8536	0.7172
**50 C**	**AUC**	0.9904	0.9068	0.7597	0.9896	0.9072	0.7628	0.9907	0.9077	0.7622	0.9868	0.8965	0.7541
	**ACC**	0.9524	0.8332	0.7006	0.9508	0.8348	0.7016	0.9536	0.8344	0.7040	0.9430	0.8238	0.6994
	**F1**	0.9526	0.8361	0.7243	0.9509	0.8367	0.7258	0.9536	0.8373	0.7253	0.9434	0.8263	0.7203
	**PREC**	0.9507	0.8350	0.6844	0.9523	0.8446	0.6980	0.9604	0.8472	0.7111	0.9539	0.8337	0.7119
	**REC**	0.9576	0.8408	0.7528	0.9532	0.8216	0.7164	0.9524	0.8356	0.6908	0.9424	0.8208	0.6784
**75 A**	**AUC**	0.9658	0.8516	0.7009	0.9453	0.8217	0.6820	0.9636	0.8485	0.6996	0.9605	0.8520	0.7045
	**ACC**	0.9012	0.7808	0.6554	0.8764	0.7524	0.6426	0.8996	0.7752	0.6560	0.8960	0.7792	0.6570
	**F1**	0.9009	0.7874	0.7017	0.8784	0.7637	0.6934	0.9019	0.7822	0.7007	0.8975	0.7858	0.7037
	**PREC**	0.9197	0.7803	0.6598	0.8711	0.7567	0.6385	0.8927	0.7834	0.6552	0.9093	0.7903	0.6721
	**REC**	0.8920	0.7856	0.6688	0.8888	0.7472	0.6668	0.9132	0.7668	0.6948	0.8988	0.7720	0.6524
**75 B**	**AUC**	0.9995	0.9643	0.8215	0.9994	0.9645	0.8196	0.9995	0.9652	0.8221	0.9989	0.9641	0.8230
	**ACC**	0.9914	0.9052	0.7492	0.9892	0.9046	0.7484	0.9906	0.9066	0.7510	0.9876	0.9040	0.7528
	**F1**	0.9914	0.9066	0.7641	0.9893	0.9059	0.7611	0.9906	0.9075	0.7629	0.9876	0.9048	0.7652
	**PREC**	0.9936	0.8990	0.7654	0.9861	0.9111	0.7510	0.9900	0.9126	0.7603	0.9892	0.9061	0.7532
	**REC**	0.9928	0.9184	0.7452	0.9944	0.9020	0.7560	0.9924	0.9164	0.7468	0.9864	0.9060	0.7560
**75 C**	**AUC**	0.9804	0.8741	0.7227	0.9883	0.9007	0.7487	0.9884	0.9017	0.7491	0.9788	0.8826	0.7363
	**ACC**	0.9300	0.7990	0.6692	0.9524	0.8258	0.6876	0.9512	0.8274	0.6902	0.9318	0.8108	0.6818
	**F1**	0.9301	0.8088	0.7023	0.9526	0.8294	0.7174	0.9519	0.8326	0.7158	0.9326	0.8134	0.7090
	**PREC**	0.9357	0.7998	0.6844	0.9549	0.8450	0.7198	0.9410	0.8228	0.7081	0.9227	0.8276	0.6959
	**REC**	0.9308	0.8216	0.6844	0.9568	0.8016	0.6332	0.9664	0.8444	0.6740	0.9436	0.7956	0.6552
**100 A**	**AUC**	0.9863	0.8776	0.7311	0.9880	0.8819	0.7356	0.9887	0.8847	0.7379	0.9843	0.8758	0.7347
	**ACC**	0.9470	0.8002	0.6790	0.9530	0.8040	0.6828	0.9528	0.8080	0.6820	0.9414	0.7996	0.6776
	**F1**	0.9472	0.8069	0.7110	0.9535	0.8117	0.7141	0.9533	0.8125	0.7149	0.9420	0.8072	0.7108
	**PREC**	0.9456	0.8035	0.6708	0.9473	0.7960	0.6727	0.9459	0.8072	0.6845	0.9343	0.8073	0.6828
	**REC**	0.9508	0.8040	0.7256	0.9636	0.8320	0.7172	0.9624	0.8176	0.6880	0.9512	0.7888	0.7004
**100 B**	**AUC**	0.9995	0.9639	0.8423	0.9988	0.9561	0.8315	0.9995	0.9682	0.8535	0.9984	0.9539	0.8308
	**ACC**	0.9922	0.9066	0.7690	0.9880	0.8918	0.7578	0.9926	0.9164	0.7782	0.9852	0.8940	0.7556
	**F1**	0.9922	0.9080	0.7849	0.9880	0.8936	0.7765	0.9926	0.9164	0.7920	0.9852	0.8957	0.7726
	**PREC**	0.9901	0.8984	0.7628	0.9888	0.8898	0.7436	0.9916	0.9194	0.7627	0.9857	0.8838	0.7572
	**REC**	0.9952	0.9220	0.7872	0.9916	0.9076	0.7940	0.9956	0.9156	0.8108	0.9868	0.9084	0.7704
**100 C**	**AUC**	0.9986	0.9538	0.8174	0.9976	0.9456	0.8036	0.9977	0.9459	0.8039	0.9979	0.9475	0.8127
	**ACC**	0.9850	0.8888	0.7524	0.9808	0.8794	0.7396	0.9810	0.8790	0.7398	0.9796	0.8820	0.7466
	**F1**	0.9850	0.8895	0.7636	0.9809	0.8799	0.7537	0.9811	0.8814	0.7534	0.9796	0.8848	0.7559
	**PREC**	0.9892	0.8944	0.7627	0.9786	0.8855	0.7291	0.9794	0.8819	0.7241	0.9831	0.8714	0.7445
	**REC**	0.9864	0.8888	0.7616	0.9856	0.8744	0.7640	0.9844	0.8788	0.7852	0.9772	0.9060	0.7564

## Data Availability

For additional details on the experimental setup, datasets, and reproducibility, the complete implementation is available in [39].

## Appendix A. Fidelity Algorithms

The following outlines the two fidelity algorithms described in Section 6.1.

**Fidelity across signals:** The first algorithm obtains a Euclidean distance-based score between two datasets of EM emissions. This is used to compare the fidelity of synthetic samples to real baseline counterparts. As shown in Algorithm A1, we first extract the CCWs from both the baseline and comparison datasets (⑨ and ⑩). Then, for each signal in the comparison dataset, we compute its Euclidean distance to every signal in the baseline dataset (⑫ to ⑯). The average of these distances is then computed and stored in a list (⑰). This process is repeated for all signals in the comparison dataset, resulting in a list of average distances that represent the signal-level similarity. Finally, this list is averaged once more to yield a single fidelity distance value that quantifies the overall similarity between the comparison and baseline datasets (⑲). This procedure is performed for each training program, and the resulting fidelity values are summed to represent the total fidelity across all training programs.

**Algorithm A1** Fidelity calculation across signals
1: **function** CCWs:(dataset of signals *S*): 2: $S_{\mathrm{CCWs}}\leftarrow \left[\;\right]$ 3: **for all** s∈S **do** 4: $S_{\mathrm{CCWs}}\leftarrow \mathrm{OBTAIN}\text{\_}\mathrm{CCW}\left(s\right)$ 5: **end for** 6: **return** $S_{\mathrm{CCWs}}$ 7: **end function** 8: **function** Fidelity_Emissions:(baseline dataset *X*, set of signals to compare *C*): 9: $X_{\mathrm{CCWs}}\leftarrow \mathrm{CCWs}\left(X\right)$ 10: $C_{\mathrm{CCWs}}\leftarrow \mathrm{CCWs}\left(C\right)$ 11: $D_{\mathrm{Emissions}}\leftarrow \left[\;\right]$ 12: **for all** $c\in C_{\mathrm{CCWs}}$ **do** 13: D←[] 14: **for all** $x\in X_{\mathrm{CCWs}}$ **do** 15: D←DISTANCE(x,c) 16: **end for** 17: $D_{\mathrm{Emissions}}\leftarrow \mathrm{AVG}\left(D\right)$ 18: **end for** 19: **return** $\mathrm{AVG}(D_{\mathrm{Emissions}})$ 20: **end function**

**Fidelity across cycles:** The following is a custom method that determines how many cycles fall within a defined normal range, as outlined in Algorithm 2. First, we extract the CCWs from both the baseline and comparison datasets, each containing EM samples from a specific update program (⑨ and ⑩). Then, we isolate the CCWs corresponding to a particular instruction cycle to perform a focused comparison between datasets (⑪ to ⑫). Following this, we compute the distance between each cycle in the comparison dataset and the corresponding cycles in the baseline dataset, similar to the approach used in the previous evaluation. The resulting list of distances per cycle is then averaged (⑲). This process is applied to both real captured emissions and synthetically generated emissions, producing two sets of distance values relative to the baseline (㉔ to ㉕). We then extract the minimum and maximum values from the real-to-baseline comparison to establish a normalcy range. Finally, we calculate the percentage of synthetic-to-baseline distances that fall within this normalcy range. The reported result reflects the percentage of cycles across all update programs that conform to this defined baseline norm. The size of the real, synthetic, and baseline datasets was 800 samples for each comparison.

**Algorithm A2** Percent of cycles within the norm
1: **function** CCWCycles:(dataset of convex center windows $W_{\mathrm{CCWs}}$, index of cycle *i*): 2: $W_{\mathrm{CCWCycles}}\leftarrow \left[\;\right]$ 3: **for all** $w\in W_{\mathrm{CCWs}}$ **do** 4: $W_{\mathrm{CCWCycles}}\leftarrow \mathrm{OBTAIN}\text{\_}\mathrm{CCW}\text{\_}\mathrm{FOR}\text{\_}\mathrm{CYCLE}\left(w,i\right)$ 5: **end for** 6: **return** $W_{\mathrm{CCWCycles}}$ 7: **end function** 8: **function** CycleDists:(baseline dataset *X*, set of signals to compare *C*, index of cycle *i*): 9: $X_{\mathrm{CCWs}}\leftarrow \mathrm{CCCWs}\left(X\right)$ 10: $C_{\mathrm{CCWs}}\leftarrow \mathrm{CCWs}\left(C\right)$ 11: $X_{\mathrm{CCWCycles}}\leftarrow$ CCWCycles(X_CCWs_) 12: $C_{\mathrm{CCWCycles}}\leftarrow$ CCWCycles(C_CCWs_) 13: CycleDist←[] 14: **for all** $c\in C_{\mathrm{CCWCycles}}$ **do** 15: D←[] 16: **for all** $x\in X_{\mathrm{CCWCycles}}$ **do** 17: D←DISTANCE(x,c) 18: **end for** 19: CycleDist←AVG(D) 20: **end for** 21: **return** CycleDist 22: **end function** 23: **function** Fidelity_Cycle:(baseline dataset *X*, set of real signals *R*, set of synthetic signals *S*, index of cycle *i*): 24: $R_{\mathrm{CycleDists}}\leftarrow$ CycleDists(*X*, *R*, *i*) 25: $S_{\mathrm{CycleDists}}\leftarrow$ CycleDists(*X*, *S*, *i*) 26: $minimum\leftarrow \min(R_{\mathrm{CycleDists}})$ 27: $maximum\leftarrow \max(R_{\mathrm{CycleDists}})$ 28: $amount\leftarrow \left|\left\{s\in S_{\mathrm{CycleDists}}|minimum\leq s\leq maximum\right\}\right|$ 29: **return** $amount/\left|S_{\mathrm{CycleDists}}\right|$ 30: **end function**

## Appendix B. Detailed Network Architectures

The generator inputs for both GAN architectures are derived from a structured Execution State Descriptor (ESD), which combines tokenized instructions, operand values, register-state information, and execution-cycle indices. Instruction mnemonics for the target instruction and up to three preceding instructions are tokenized using the TensorFlow Keras Tokenizer, which assigns each mnemonic a unique integer index while preserving program order (i.e., $\left(Inst_{t-3},Inst_{t-2},Inst_{t-1},Inst_{t}\right)$). Operand values are then appended by extracting the values stored in the referenced registers at the moment when each instruction executes. For example, if an instruction add r16, r17 executes when r16=30 and r17=55, the operand pair (30, 55) is added to the descriptor. This is repeated for each instruction in the window, thus capturing the evolution of register values over time. To incorporate full device state, the complete register file (R0,R1,…,R31) of the ATmega2560 is appended prior to the target instruction. Finally, because AVR instructions may span multiple clock cycles, the cycle index of the EM measurement is included as the final element of the ESD.

To ensure controlled comparisons between architectures, the 1DCNNGAN and ResGAN models were trained under a unified CWGAN-GP configuration. Both models were conditioned on one- to four-instruction ESD sequences containing instruction tokens, operand values, and register states. Training used the Adam optimizer with parameters $\left(\beta_{1}=0.5,\;\beta_{2}=0.9\right)$ and a learning rate of $1\times 10^{-4}$ and followed the standard WGAN-GP regime of performing five discriminator updates per generator update. The gradient penalty coefficient was set to λ=10 to enforce the Lipschitz constraint. All layers employed He-normal initialization with LeakyReLU activations (α=0.2). Training proceeded for 50,000 generator iterations, with convergence monitored through CCW fidelity rather than epoch count. Before training, all EM traces were normalized, and each ESD was constructed by concatenating code tokens, operand values, full-register state, and cycle index—in that order. These settings were applied consistently across both models to isolate architectural effects on generative performance.

Beyond defining the ESD inputs, the following subsections provide a detailed description of the two generative architectures evaluated in this work: the lightweight 1DCNNGAN model and the deeper ResGAN baseline. Each subsection outlines the design rationale, layer configuration, and computational characteristics of the respective models, enabling a transparent comparison of their capabilities in EM waveform synthesis. While these summaries capture the architectural structure, the exact implementation—including model construction, training loops, preprocessing routines, and evaluation scripts—is fully documented in the accompanying Jupyter notebooks. For complete reproducibility, readers are referred to the publicly available code repository [39].

### Appendix B.1. 1DCNNGAN Architecture

The 1DCNNGAN model is a lightweight convolutional generative architecture tailored for EM waveform synthesis from structured ESD inputs. After reshaping the input vector into a sequence format, the generator applies two 1D convolutional layers (128 and 256 filters) to extract localized temporal features. Each convolutional block is followed by LeakyReLU activation and batch normalization to stabilize WGAN-GP training. The resulting feature map is flattened and projected through fully connected layers to produce the final waveform sample. The complete generator specification is shown in Listing  A1.

**Listing A1:** Summary of 1DCNNGAN generator.

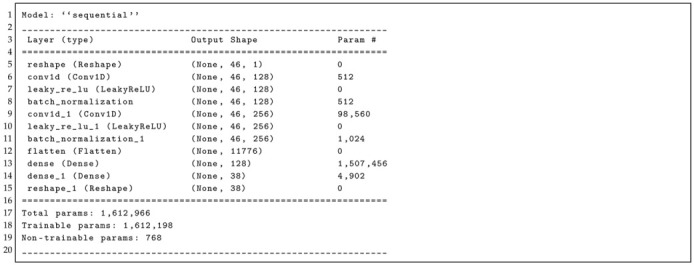

The discriminator complements this lightweight design through a deep fully connected architecture that evaluates waveform realism under the WGAN-GP objective. It consists of five dense layers of 500 units, each followed by LeakyReLU activation and batch normalization, concluding with a single output neuron representing the critic score. A dropout layer is applied before the output to improve robustness. Listing A2 outlines the full 1DCNNGAN discriminator.

**Listing A2:** Summary of 1DCNNGAN discriminator.

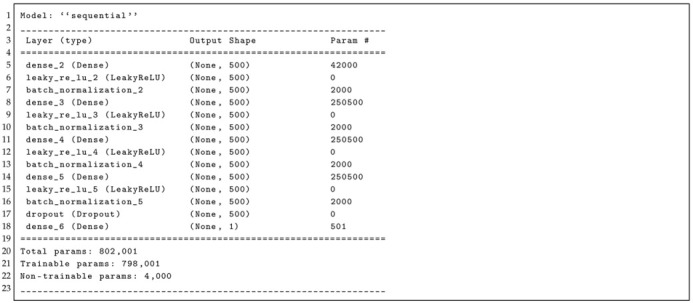

Together, these components form a compact yet expressive GAN architecture suited for time-series EM synthesis. Its combination of convolutional feature learning and dense-layer discrimination provides stable WGAN-GP training behavior while maintaining computational efficiency.

### Appendix B.2. ResGAN Architecture

ResGAN provides a deeper baseline architecture by incorporating a full ResNet-50 backbone into the generator. As with 1DCNNGAN, structured ESD inputs are first reshaped, but the representation is then expanded through a small 1D convolutional layer before being processed by the ResNet-50 module (with ImageNet classification layers removed). Residual connections in the ResNet-50 block improve gradient flow and support the learning of complex waveform structures. After feature extraction, global average pooling reduces the representation to a compact embedding, which is passed through dense layers to generate the final EM output. The full ResGAN generator is depicted in Listing A3.

**Listing A3:** Summary of ResGAN generator.

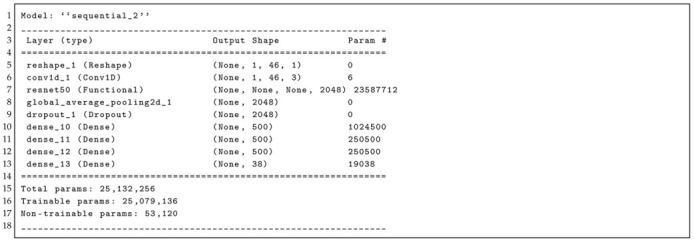

The ResGAN discriminator mirrors the deep fully connected structure of 1DCNNGAN’s discriminator but contains a larger parameter count to match the expressive power of the ResNet-based generator. Five sequential dense layers of 500 units process the waveform before the final critic output. An overview of the discriminator model is listed in Listing A4.

**Listing A4:** Summary of ResGAN discriminator.

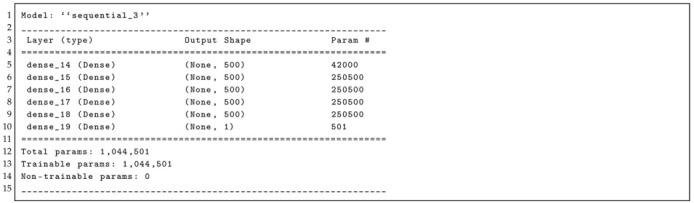

Overall, the ResGAN architecture emphasizes depth and residual learning, offering a rich representational capacity for EM waveform generation. Its comparison with the lightweight 1DCNNGAN model highlights the tradeoff between computational efficiency and expressive power. The detailed specifications provided here ensure full reproducibility of both models.
